# Potential strengths and limitations of the Better at Home Program identified by professionals and managers: a scoping review of the literature^*^

**DOI:** 10.1590/1518-8345.8251.4879

**Published:** 2026-07-24

**Authors:** Cristina Nunes Viana, Leticia Alves da Silva e Silva, Fernanda Cirne Lima Weston, Adriana Aparecida Paz

**Affiliations:** 1 Universidade Federal de Ciências da Saúde de Porto Alegre, Porto Alegre, RS, Brazil.; 2 Prefeitura Municipal de Porto Alegre, Secretaria Municipal de Saúde, Porto Alegre, RS, Brazil.; 3 Pontifícia Universidade Católica do Rio Grande do Sul, Porto Alegre, RS, Brazil.; 4 Novocure, DSS Learning and Operations Specialist, Porto Alegre, RS, Brazil.; 5 Scholarship holder at the Coordenação de Aperfeiçoamento de Pessoal de Nível Superior (CAPES), Brazil.; 6 Scholarship holder at the Conselho Nacional de Desenvolvimento Científico e Tecnológico (CNPq), Brazil.

**Keywords:** Home Care Services, Hospital-Based Home Care Services, Home Nursing, Home Health Nursing, Homebound Persons, Home Schooling.

## Abstract

**Objective:**

to map the strengths and limitations of the Better at Home Program identified by health professionals and managers in the scientific literature.

**Method:**

this is a scoping review based on the JBI manual and the PRISMA-ScR guidelines. The search was conducted in the MEDLINE/PubMed, LILACS, Scopus, Embase, Web of Science, CINAHL databases, and CAPES theses and dissertations, covering publications between 2011 and 2024.

**Results:**

552 studies were identified, eight met the inclusion criteria and were analyzed. The program’s potential lies in promoting dehospitalization, strengthening networking, developing educational health initiatives with families, and providing flexibility in care management. On the other hand, limitations were evident in relation to insufficient continuing education for professionals, emotional exhaustion among teams, technological limitations, and difficulties in communicating with the Health Care Network.

**Conclusion:**

the Better at Home Program is a relevant strategy in the dehospitalization process; however, it faces structural and operational challenges that compromise its sustainability. Investments in technological support and team strengthening are necessary to improve the continuity and quality of home care. Study registry: Open Science Framework: DOI 10.17605/OSF.IO/6UCDB

## Highlights

**(1)** Strengthening network care promotes safe dehospitalization. **(2)** Health education promotes autonomy among users and caregivers. **(3)** The use of telecare expands access and improves the quality of home care. **(4)** Continuing health education is essential for the program’s effectiveness. **(5)** Lack of structure and emotional support weaken home care teams.

## Introduction

Home care (HC) is a type of care provided in the user’s home, which can be carried out within the scope of Primary Health Care (PHC) or, in cases that require more complex care, through the Better at Home Program (PMeC). In recent years, HC has gained increasing relevance in the Unified Health System (SUS), especially because it provides continuous, humanized care focused on the needs of users. Integrated into the Health Care Network (HCN), this type of care stands out for offering assistance in the home environment, prioritizing the safety and well-being of users^1^.

HC promotes treatment, rehabilitation, palliative care, and prevention measures, and is recommended for users who are temporarily or permanently confined to bed or home due to clinical conditions or situations of vulnerability^2^. This type of care recognizes the social and cultural context of the user and their family members, contributing to reducing the risk of infections, repeat hospitalizations, and inappropriate use of hospital services. In addition, it promotes the efficient management of hospital beds and available health resources, serving as a qualified “exit door” from the urgent and emergency care network, with the potential to reduce overcrowding in these services^3^.

The PMeC focuses on the dehospitalization of users who require clinical monitoring and support from a multidisciplinary team, promoting not only care, but also educational activities for users, family members, guardians, and caregivers to ensure continuity of care at home. The PMeC operates through Home Care Services (HCS), which complement primary health care and emergency services and can also be a substitute for hospitalization^2^.

In view of the aging population and the growing demand for more comprehensive and humanized healthcare, HC services have been expanding worldwide^4^. In Brazil, HCSs began to be structured more intensively in 1990 and were strategically and definitively incorporated into the SUS in 2011 with the creation of the PMeC^5^.

Factors such as accelerated demographic and epidemiological transition, rising healthcare costs, the process of deinstitutionalization, and the search for more humanized models of care highlight the importance of expanding the PMeC nationwide^3^ ^-^ ^5^. To improve its management, it is important to understand the perceptions of professionals and managers about the program in terms of its challenges and potential.

The multidisciplinary teams working in the PMeC experience different barriers and challenges in the practice of providing care to users and are therefore a valuable source for identifying elements that can support improvements in the program’s management. However, despite the relevance of their experiences, no reviews have been identified in the scientific literature that bring together these perceptions of the daily routine of the PMeC since its creation. In this context, this review aimed to map the strengths and limitations of the Better at Home Program identified by health professionals and managers in the scientific literature.

## Method

### Type of study

This is an evidence-based literature review of the scoping review type, guided by the recommendations of the JBI Reviewer’s Manual, which provides guidance on mapping key concepts, clarifying areas of research, and identifying gaps in knowledge^6^. Five stages proposed by the JBI were followed in conducting the study: 1) Formulation of the research question; 2) Identification of relevant studies; 3) Selection of studies; 4) Data extraction; and 5) Synthesis and presentation of results. The research protocol was registered on the Open Science Framework platform under DOI 10.17605/OSF.IO/6UCDB^7^. The Preferred Reporting Items for Systematic Reviews and Meta-Analyses for Scoping Reviews (PRISMA-ScR) extension was used to report transparently on the conduct of the scoping review results^8^. The study was conducted in the city of Porto Alegre, in the state of Rio Grande do Sul, Brazil, from February 2024 to March 2025.

### Selection criteria

The inclusion criteria were: studies published in full, available electronically, without language restrictions, that answered the proposed research question. The time frame was set from 2011 to 2024, due to the approval of the PMeC Ordinance by the Ministry of Health (MS) in 2011^9^, thus covering thirteen years of program implementation in Brazil. Editorials, letters to the editor, websites, news, and summaries of scientific events were excluded.

### Data collection

The PCC mnemonic structure was adopted for the review search strategy, considering: Population (P) - health professionals and managers; Concept (C) - strengths and limitations of the PMeC; and Context (C) - Brazil. The question formulated was: “What are the strengths and limitations observed by health professionals and managers regarding the Better at Home Program in Brazil?”

The search strategy included terms present in the PCC structure, using descriptors identified in health thesauri such as Medical Subject Headings (MeSH) and Health Sciences Descriptors (DeCS). As this is a program exclusive to the Brazilian context, the research was supported by a librarian in the construction of the search strategy, who suggested using the program’s own name in Portuguese, “*Programa Melhor em Casa*”, and in English, “Better at Home Program”, in order to have greater coverage of the bibliography on the subject and expand the search strategy (Figure 1).

The search for scientific evidence was conducted in the following data sources: Latin American and Caribbean Health Sciences Literature (LILACS); Public Medical Literature Analysis and Retrieval System Online (MEDLINE/PubMed); Scopus; Excerpta Medica data BASE (EMBASE); Web of Science (WoS); and Cumulative Index to Nursing and Allied Health Literature (CINAHL). Gray literature was searched in the Brazilian Digital Library of Theses and Dissertations of CAPES.

**Figure 1:** Search strategies and databases used in the literature review. Porto Alegre, RS, Brazil, 2025

Database	Search strategy
LILACS*	(“melhor em casa” OR “programa melhor em casa”) AND programa AND (year_cluster:[2011 TO 2024]) AND ( db:(“LILACS”)) AND (year_cluster:[2011 TO 2024])
MEDLINE/PubMed	(“Better at home” OR “Better at home program”) AND program AND (brazil OR brazilian) Filters: from 2011 - 2024 (((“better”[All Fields] AND (“home environment”[MeSH Terms] OR (“home”[All Fields] AND “environment”[All Fields]) OR “home environment”[All Fields] OR “home”[All Fields])) OR (“better”[All Fields] AND (“home environment”[MeSH Terms] OR (“home”[All Fields] AND “environment”[All Fields]) OR “home environment”[All Fields] OR “programmable”[All Fields] OR “programmably”[All Fields] OR “programme”[All Fields] OR “programme s”[All Fields] OR “programmed”[All Fields] OR “programmer”[All Fields] OR “programmer s”[All Fields] OR “programmers”[All Fields] OR “programmes”[All Fields] OR “programming”[All Fields] OR “programmings”[All Fields] OR “programs”[All Fields]))) AND (“program”[All Fields] OR “program s”[All Fields] OR “programe”[All Fields] OR “programed”[All Fields] OR “programes”[All Fields] OR “programing”[All Fields] OR “programmability”[All Fields] OR “programmable”[All Fields] OR “programmably”[All Fields] OR “programme”[All Fields] OR “programmes”[All Fields] OR “programmed”[All Fields] OR “programmer”[All Fields] OR “programmers”[All Fields] OR “programmers”[All Fields] OR “programmes”[All Fields] OR “programming”[All Fields] OR “programmings”[All Fields] OR “programs”[All Fields]) AND (“brazil”[MeSH Terms] OR “brazil”[All Fields] OR “brazil”[All Fields] OR”home”[All Fields]) AND (“program”[All Fields] OR “programs”[All Fields] OR “programe”[All Fields] OR “programed”[All Fields] OR “programes”[All Fields] OR “programing”[All Fields] OR “programmability”[All Fields] OR “brazil”[All Fields] OR (“brazilian people”[Supplementary Concept] OR “brazilian people”[All Fields] OR “brazilians”[All Fields] OR “brazilian”[All Fields]))) AND (2011:2024[pdat])
Scopus	TITLE-ABS-KEY ( ( “Better at home” OR “Better at home program” ) AND program ) AND PUBYEAR > 2010 AND PUBYEAR < 2024
EMBASE	(‘better at home’ OR ‘better at home program’) AND ‘program’ AND [2011-2024]/py
WoS^†^	(“Better at home” OR “Better at home program”) AND program (All Fields) and 2018 or 2019 or 2023 or 2022 (Publication Years)
CINAHL	(‘better at home’ OR ‘better at home program’) AND ‘program’
CAPES Catalog of Theses and Dissertations	“melhor em casa”

*LILACS = Latin American and Caribbean Literature in Health Sciences; ^†^WoS = Web of Science

### Selection of studies

All records retrieved from the databases were identified, grouped, and uploaded to the Rayyan^®^ (Intelligent Systematic Review) application, where duplicates were removed and initial screening of titles and abstracts was performed^10^. The gray literature was analyzed in Excel^®^ spreadsheets, also through initial reading of the title and abstract. After removing duplicates in Rayyan^®^, the initial stage of data analysis was performed, with reviewers conducting independent and blind reading of titles and abstracts of all documents on the Rayyan^®^ platform and in the Excel^®^ spreadsheet. The reviewers assigned acceptance or rejection concepts according to the review’s research question. The blinding of the Rayyan^®^ platform was opened in a meeting between the reviewers to verify the discrepancies. In this sense, the studies that remained with discrepancies were evaluated by a third reviewer, who performed the evaluation on the Rayyan^®^ platform. Regarding the grey literature, a face-to-face meeting was held with the two reviewers to evaluate the results and discuss the discrepancies, so that the intervention of a third reviewer was not necessary.

The selected studies were read in full and analyzed descriptively in accordance with the research question and the relevance of the study objective. Regarding the grey literature, one specific dissertation was not found in its entirety. Attempts to contact the author and advisor by email were unsuccessful, and the other dissertation was the result of an article selected in the study, so it was decided to use the published article.

### Data processing

Based on the selected documents, the extracted data were organized in an Excel^®^ spreadsheet containing information on: author, title, type of study, year of publication, language, sample, publication journal, country of origin, year of publication, study objectives, main results, and conclusions. The data were analyzed descriptively, and the findings were subsequently categorized into strengths and limitations of the PMeC through a thematic analysis.

### Ethical aspects

As this was a scoping review, there was no need to submit the study to the Research Ethics Committee. It is stated that there was no conflict of interest on the part of the researchers involved and that the copyright of the documents cited was respected in accordance with Law No. 12,853 of 2013^11^.

## Results

**Figure 2:**
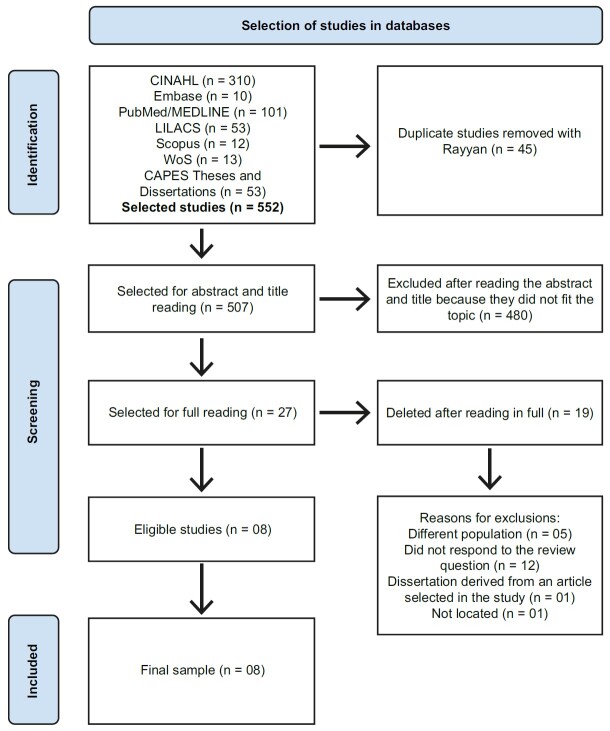
PRISMA-ScR^12^ flowchart used for study identification and selection. Porto Alegre, RS, Brazil, 2025

In the database search, 552 publications were identified. After removing 45 duplicates, 507 studies remained. Next, the titles and abstracts were read, and the inclusion and exclusion criteria were applied by two independent reviewers, resulting in the selection of 27 studies. Subsequently, the selected documents were read in full, considering the established inclusion and exclusion criteria, culminating in a final sample consisting of eight publications (Figure 2).

The main language of the publications was Portuguese (n=5; 62.5%), followed by English (n=3; 37.5%). Regarding the methodological design used by the authors, qualitative studies prevailed (n=6; = 75%), followed by quantitative studies (n=1; 12.5%) and mixed methods (n=1; 12.5%).

A total of 90 participants were identified in the studies. Regarding the year of publication, the articles were published between 2016 and 2022, with the highest number in 2022 (n=3; 37.5%), followed by 2018 (n=2; 25%), 2020 (n=1; 12.5%), 2019 (n=1; 12.5%), and 2016 (n=1; 12.5%). Figure 3 presents the characterization of the studies included in the review, with information on authorship, title, type of study, year of publication, and number of participants.

**Figure 3:** Characterization of the studies identified in the review. Porto Alegre, RS, Brazil, 2025

Study	Author	Title	Type of study	Year of publication	Sample (n=)
E1^13^	Verdi DS, Pires RRC	Between standardization and flexibility: the implementation of federal program Better at Home considering local diversity	Descriptive study with a qualitative approach	2022	6
E2^14^	Almeida MB, Albuquerque IMAN, Silva MAM, Mayorga FDO, Balica HLL, Nascimento ABO	Limits and Potentials of Pediatric Home Care in a Ceará State municipality	Evaluative study with a qualitative approach	2022	-
E3^15^	Cavalcante MEPL, Santos MM, Toso BRGO, Vaz EMC, Lima PMVM, Collet N	It is better at home: characterization of home care services	Exploratory study quantitative	2022	23
E4^16^	Castro EAB, Leone DRR, Santos CM, Neta FCCG, Gonçalves JRL, Contim D, Silva KL	Home care organization with the Better at Home Program	Descriptive, exploratory study with a qualitative approach	2018	18
E5^17^	Lima ACB	*Análise da implantação do serviço de atendimento domiciliar na óptica dos profissionais da atenção primária à saúde*	Descriptive, exploratory study with a qualitative approach	2020	14
E6^18^	Maldonado TCP	*“Melhor em Casa!?” A resiliência do profissional frente a prática do atendimento domiciliar*	Multiple case study with a qualitative approach	2019	9
E7^19^	Oliveira AV Neto	*Análise do Programa Melhor em Casa: um olhar sobre a atenção domiciliar no âmbito do Sistema Único de Saúde*	Descriptive, exploratory study quantitative and qualitative approach	2016	6
E8^20^	Canuto KF	*Melhor em Casa: um estudo de caso sobre a interdisciplinaridade no programa no município de Palmeira dos Índios, Alagoas*	Evaluative study with a qualitative approach	2018	14

Figure 4 summarizes the objectives of the studies and the main results of each study, allowing us to visualize the perceptions of professionals regarding the PMeC.

**Figure 4:** Descriptive summary of the studies included in the scoping review. Porto Alegre, RS, Brazil, 2025

Study	Objectives	Key insights from professionals
E1^13^	Learn about the different experiences of local managers with federal guidelines and regulations related to the implementation of the Better at Home Program.	In terms of potential, the program’s guiding ordinance is considered appropriate to the reality. The importance of federal coordination instruments with local adaptations and the need for flexibility in the recommendations of the PMeC* ordinance are recognized. Participation in the program has brought greater financial resources to HC^†^, improving the health team and the flow of care. The PMeC* has allowed deaths to occur at home and has led to dehospitalization. However, there is still resistance on the part of hospital medical staff to refer patients to the program, as well as difficulties in the composition of HC^†^ teams.
E2^14^	Understanding the limitations and potential of pediatric home care provided by the Better at Home Program in a municipality in the state of Ceará.	The lack of knowledge among professionals at different levels of care about HC^†^ hinders continuity of care, as does the absence of certain professional categories, the lack of dedicated physical infrastructure for PMeC*, and the shortage of essential care supplies. There was a lack of transportation, training, and links between the program and PHC^‡^ management. Another negative feature mentioned was the change of professionals at each management cycle, severing the link between professionals, patients, and the network. However, one criterion for improvement is the decrease in pediatric hospital admissions.
E3^15^	Characterizing Home Care Services operating in Paraíba.	The use of call centers is a major strategy for user care. Areas for improvement include HCS^§^ transportation, which is shared with other services in the network. Some teams do not meet the minimum required working hours, working only certain shifts, which affects the continuity of care. HCS^§^ headquarters are usually attached to other health services, and even with interconnection, there is fragmentation in communication between services. With regard to electronic patient records and ITP^||^, these are only carried out in 50% of services, demonstrating fragmentation in some essential areas for patient information and individualized care planning.
E4^16^	Understanding the organization of Home Care in the context of public health care provided by municipalities that have joined the Better at Home Program in the State of Minas Gerais.	They perceive integration with the network as a weakness of the PMeC*. Holding meetings between teams from different services and organizing processes are seen as effective strategies for improving user service in HC^†^.
E5^17^	Analyze how the Home Care Service was implemented from the perspective of primary health care professionals in the municipality of Araranguá, Santa Catarina.	The importance of the PMeC* in the dehospitalization of patients and in improving the quality of life of users and their families has been highlighted, serving as a reference for PHC^‡^ and hospitals. Areas for improvement include the lack of care sharing, the difficulty of interaction between professionals, and the lack of knowledge of referrals by PHC^‡^. In addition, there is an overload of the HCS^§^ team, a lack of physical structure and vehicles, and difficulty in holding users accountable for care. Continuing education in health with HCN^¶^ professionals is suggested.
E6^18^	Understanding the experience of professionals in the Better at Home Program in light of resilience.	The PMeC* is seen as a welcoming, decisive service that performs humanized, multidisciplinary work, looking beyond clinical demands. The service provides support and care to families, making it a unique service in the HCN^⁋^. The service enters homes and, because it has such close contact with diverse situations, professionals are exposed to overload, helplessness, and fragility. Due to the strong bond with families, some are reluctant to be discharged from the PMeC*.
E7^19^	Characterize the implementation of the Better at Home Program and analyze it from the perspective of municipal managers.	The PMeC* is perceived as an alternative to hospitalization, has great potential for dehospitalization and coordination with the HCN^¶^. It optimizes hospital beds, enters the network for case discussion and care sharing. Greater attention should be given to the dissemination of the program by the Ministry of Health to the population, with a need for further explanation of the ordinance with the HCN^¶^ for better understanding. Incentives to improve the technical quality of the PMeC* team and the correct supply of inputs and training to PHC^‡^ for the proper referral of patients.
E8^20^	Assess professionals’ understanding of interdisciplinarity in the actions of the Better at Home Program in the municipality of Palmeira dos Índios, Alagoas.	The meaning of interdisciplinarity is still little known or misunderstood among professionals. At HCS^§^, it occurs through everyday actions, in horizontal relationships with exchanges or shared care. Teamwork is closely linked to the lack of vehicles exclusively for HCS^§^, or the use of an exclusive vehicle for dressings, demonstrating a lack of shared care. Admissions are carried out only by a social service professional, where no exchanges are made, and ITP^||^ is not performed. There is a need for improvements in interpersonal relationships, team meetings, multiprofessional care, as well as the dissemination of program criteria for HCN^¶^.

*PMeC = Better at Home Program; ^†^HC = Home Care; ^‡^PHC = Primary Health Care; ^§^HCS = Home Care Service; ^||^ITP = Individualized Treatment Plan; ^¶^HCN = Health Care Network

To facilitate understanding and organization of the results, as well as to answer the review’s research question, the findings were subdivided into two thematic categories: Strengths and Limitations of the PMeC, as shown in Figure 5.

**Figure 5:** Thematic categories of the scoping review. Porto Alegre, RS, Brazil, 2025

Thematic Categories
Strengths of the Better at Home Program
Building networks^13^ ^-^ ^14^ ^)(^ ^16^ ^),(^ ^18^ ^-^ ^20^; Integration of the service with society^13^; Organizational protocols and processes^16^; Guidelines appropriate to reality^13^; Dehospitalization and reduction of unnecessary hospitalizations^13^ ^-^ ^14^ ^),(^ ^17^; Health education with families^14^ ^),(^ ^16^ ^-^ ^17^; Call center^15^; Individualized Treatment Plan (ITP)^16^; Flexibility and autonomy on the part of management^20^; Humanization^15^; and Improvement in users’ quality of life^17^.
**Limitations of the Better at Home Program**
Continuing education^14^ ^-^ ^20^; Dialogue with HCN*^13^ ^-^ ^19^; Structuring of services^13^ ^-^ ^17^ ^),(^ ^20^; User responsibility for care^19^ ^-^ ^20^; Emotional exhaustion of professionals^18^ ^-^ ^19^; Lack of technological resources^15^; Use of electronic medical records^15^; and Fragmentation of the team^20^.

*HCN = Health Care Network

## Discussion

The findings of this scoping review highlighted two thematic categories that characterize the program’s performance in the Brazilian context: the strengths and limitations of the PMeC. Critical analysis allows us to understand the progress achieved and the persistent challenges in consolidating HC as a strategy integrated into the HCN.

### Strengths for the Better at Home Program

The development of networking, as a structuring axis of the PMeC, stood out as the main potential highlighted in the studies analyzed. Coordinated action between the different points of the HCN is fundamental, considering that the program mainly assists users who have recently been hospitalized. This configuration requires coordination with the intra-hospital network to ensure a safe transition of care, the establishment of intra-family ties between the PMeC team and the family at home, and, subsequently, the integration of the user into PHC, thus creating a continuum of care in the HCN. In this context, the consolidation of networking is necessary and intrinsically related to the structure of the service, contributing to better clinical outcomes. A narrative review highlighted that the quality of home care is influenced both by the ability of professionals to address needs and by the establishment of direct communication with the family^21^. This reinforces coordination with the different levels of the HCN, which favors the formulation of strategies for integrated actions that meet, in a multidisciplinary manner, the needs and expectations of users and families in the face of uncertainties associated with home care.

Another point highlighted in the studies refers to dehospitalization and the reduction of hospitalizations considered avoidable, corroborating the central objectives of the program, which aim at the safe transition of stable users to their homes. The user’s return to the home environment is a continuation of the care initiated in the hospital context and, when carried out in a planned manner and with access to the necessary resources, allows for the maintenance of coordination with the different levels of the network in an integrated, decisive, and person-centered manner. Planning for a safe discharge involves assessing the user’s clinical and social needs, as well as their family structure and the availability of HCN services, which are fundamental elements for preventing readmissions^22^. Still in this context, the reduction of unnecessary hospitalizations is favored by the early recognition of changes in the user’s clinical condition and by the adequate management of complications, which is the result of the educational process promoted by the teams with the families, contributing to a decrease in the user’s return to the hospital environment.

Health education aimed at families was represented in the studies as one of the differential strategies of the PMeC. The program goes beyond the focus on dehospitalization by investing in educational actions with caregivers and family members in the home environment, where intense exchanges of knowledge occur. In this intimate and unique context, marked by the reorganization of the family routine in the presence of a sick family member, it is essential that the team demonstrates empathy and commitment to progressively promote the construction of knowledge among family members for the exercise of safe and comprehensive care. Health education should, therefore, be conceived as a tool for promoting the autonomy and protagonism of all those involved, encouraging co-responsibility in the care process^23^.

The adoption of telecare and ITP was also highlighted as a strategy that personalizes care, placing the user at the center of their rehabilitation process and promoting greater agility and effectiveness in care actions. Telecare is a support system that allows users to remain at home, while the platform’s visual resources enable the development of interpersonal relationships with healthcare professionals over time. This modality is a viable alternative for monitoring symptoms, guiding actions, and encouraging adherence to home care, exploring both care and educational possibilities^24^. With regard to care planning, the ITP serves as a guide for the actions of the multidisciplinary team, being shared among team members with a view to implementing interventions tailored to the unique needs of each user^25^ ^-^ ^26^.

### Limitations of the Better at Home Program

The most recurrent limitations identified in the studies were continuing education and dialogue with the HCN, highlighting two central aspects that require qualified attention in the national context. Another aspect frequently pointed out in the studies refers to the structure of services, followed by user accountability for care and the emotional exhaustion of professionals involved in HC.

Continuing education, recognized as an in-service teaching-learning strategy with problematizing, interdisciplinary, and interprofessional principles in a real context, focuses on the daily needs of professional practice to improve the quality of care provided to users, especially in the PMeC, given the complexity of home care. This educational process has brought about gradual changes in health practices, with the aim of improving care, reorganizing the work process, and enhancing the quality of public management through innovative pedagogical actions aligned with the current care model^27^. Such actions should include innovative methodologies, including virtual learning and technological resources, that promote the development of skills among healthcare workers^4^.

Fragmentation in communication with the HCN or difficulties in accessing users in the PMeC in situations of intercurrence constitute significant obstacles that lead to emotional exhaustion in multidisciplinary teams, as well as to the resolution of user needs. These barriers compromise the comprehensiveness of care and can lead to discontinuity of care, recurrence of health problems, and avoidable readmissions, often related to the fragility of coordination between the services that make up the network. Effective integration between HCN services, based on a shared vision, adoption of clinical guidelines, and horizontal organization, is imperative for improving the quality of care and reducing redundancies^28^.

The structuring of services, in turn, ranges from limitations in inadequate physical facilities to insufficient vehicles, often shared with other services or municipal units, compromising the performance of home visits. The adequate provision of material resources, although essential, is not sufficient to ensure the effectiveness of care in the home context. For HC to be carried out efficiently and safely, strategic planning is necessary that incorporates continuing education and ensures the adequate allocation of material resources and qualified personnel management^28^.

Another emerging aspect in the studies refers to the need to incorporate new technologies, such as the PEP. The absence of this resource limits the sharing of information between HCN services, hindering the continuity and comprehensiveness of care. The implementation of the PEP, in addition to providing greater practicality and agility in information management, is essential for the expansion of HC^29^. PEP contributes to the safety and effectiveness of care, allowing access to user history and supporting clinical decision-making^26^.

The accountability of users and families for care is a challenge in some studies. This aspect requires an approach from the moment the user is admitted to the program, through clear agreements with the family regarding care processes and discharge, ensuring that users and caregivers are equipped for care, in order to awaken interest, co-responsibility, and the sharing of knowledge and expertise^30^. It is necessary to promote family autonomy in the process of co-responsibility and encourage the protagonism of those involved, and consolidate HC as a reference for care whenever necessary^31^. HC is a new and innovative alternative to care provision outside traditional institutions such as hospitals, allowing the family and caregiver to take on a central role in managing care at home, contributing to the development of strategies that are more appropriate to the realities of the territory^32^.

Another aspect of vulnerability identified in two studies was the emotional exhaustion of professionals, especially when caring for users with chronic diseases. Working in the home exposes professionals to adverse realities, including poor housing conditions and scarcity of resources, conditions that increase psychological distress and emotional overload. Such conditions require managers to pay attention to the mental health of their teams through health education initiatives, listening spaces, and psychosocial support^33^. Continued exposure to psychosocial risks can result in significant physical, mental, and social impacts, often overlooked due to prejudices that associate psychological distress with individual fragility, to the detriment of understanding the working conditions involved in everyday life at HC^34^.

Among the limitations of the study, we highlight the scarce availability of scientific publications on the subject, which highlights the need for further research to gain a more comprehensive understanding of the program’s potential and identify improvements that can be applied to the PMeC. Considering the cultural and geographical diversity of the country, many actions can be adapted, recreated, and expanded. Continuing education is also a significant limitation, indicating the relevance of developing intervention studies aimed at training professionals in the PMeC.

This study makes significant contributions to nursing by offering a critical and comprehensive analysis of the PMeC, highlighting its potential and limitations in the context of HC integrated with the HCN. The role of nursing in the context of the multidisciplinary team in the PMeC is essential in coordinating networked care, educating families about health, and promoting safe, user-centered continuity of care. On the other hand, the study supports the improvement of professional practices and contributes to the strengthening of HC as an innovative and decisive strategy in terms of continuing education, the use of technologies such as telecare and electronic medical records, and the valorization of mental health care in the face of the emotional exhaustion of teams. This reinforces the strategic role of nursing in coordinating care, improving the quality of home care, and building more sustainable, efficient, and humanized care models.

## Conclusion

The mapping of scientific publications on the strengths and limitations of the PMeC, as identified by healthcare professionals and managers, proved to be limited in quantity when compared to other areas of healthcare knowledge. The strengths of the PMeC identified included networking, dehospitalization, reduction of avoidable readmissions, health education for families, telecare, use of the ITP, flexibility and autonomy in management, as well as humanization and improvement in the quality of life of users. With regard to the limitations observed, aspects that require qualified measures to improve the program and home health care stand out: continuing education, dialogue with the HCN, the structure of services, the shared responsibility of users and family members for care, the emotional exhaustion of professionals, the insufficiency of technological resources, the absence of the PEP, and the fragmentation of the team.

Thus, the study highlights the relevance of the PMeC in the Brazilian healthcare system by demonstrating its potential to improve home care. To this end, strategic interventions and structural investments are needed to strengthen the program and ensure its long-term effectiveness and sustainability. It is also recommended that further studies be conducted to explore the topics addressed in greater depth, contributing to the advancement of knowledge and the improvement of practices in the field of home care offered by the PMeC.

## Data Availability

All data generated or analysed during this study are included in this published article.

## References

[1] Ministério da Saúde (BR), Secretaria de Atenção à Saúde, Departamento de Atenção Básica (2012). Caderno de atenção domiciliar.

[2] Ministério da Saúde (BR), Gabinete do Ministro (2024). Altera as Portarias de Consolidação nos 5 e 6, de 28 de setembro de 2017, para atualizar as regras do Serviço de Atenção Domiciliar (SAD) e do Programa Melhor em Casa (PMeC). Portaria GM/MS nº 3.005, de 2 de janeiro de 2024.

[3] Savassi LCM, Dias MB, Boing AF, Verdi M, Lemos AF (2020). Educational strategies for human resources in home health care: 8 years’ experience from Brazil. Rev Panam Salud Publica.

[4] Ministério da Saúde (BR), Departamento de Atenção Hospitalar, Domiciliar e de Urgência (2023). Potencializar a utilização do Programa Melhor em Casa: relatório de análise de impacto regulatório.

[5] Colussi CF, Hellmann F, Verdi M, Serapioni M, Savassi LCM, Ferreira DD (2021). Evaluability study of the Multicenter Program for Professional Qualification in Distance Home Care (PMQPAD). Cad Saude Publica.

[6] Aromataris E, Lockwood C, Porritt K, Pilla B, Jordan Z (2024). JBI manual for evidence synthesis.

[7] Viana CN, Paz AA, Weston FCL, Silva LAS (2024). Strengths and weaknesses of the Better at Home Program: scoping review. Open Science Framework.

[8] Tricco AC, Lillie E, Zarin W, O’Brien KK, Colquhoun H, Levac D (2018). PRISMA extension for scoping reviews (PRISMA-ScR): checklist and explanation. Ann Intern Med.

[9] Ministério da Saúde (BR) (2011). Redefine a Atenção Domiciliar no âmbito do Sistema Único de Saúde (SUS). Portaria nº 2.527, de 27 de outubro de 2011.

[10] Ouzzani M, Hammady H, Fedorowicz Z, Elmagarmid A (2016). Rayyan-a web and mobile app for systematic reviews. Syst Rev.

[11] Presidência da República (BR), Casa Civil, Subchefia para Assuntos Jurídicos (2013). Altera os arts. 5º, 68, 97, 98, 99 e 100, acrescenta arts. 98-A, 98-B, 98-C, 99-A, 99-B, 100-A, 100-B e 109-A e revoga o art. 94 da Lei nº 9.610, de 19 de fevereiro de 1998, para dispor sobre a gestão coletiva de direitos autorais, e dá outras providências. Lei nº 12.853, de 14 de agosto de 2013.

[12] Page MJ, McKenzie JE, Bossuyt PM, Boutron I, Hoffmann TC, Mulrow CD (2021). The PRISMA 2020 statement: an updated guideline for reporting systematic reviews. BMJ.

[13] Verdi DS, Pires RRC (2022). Between standardization and flexibility: the implementation of federal program “Better at home” considering local diversity. Rev Serv Publico.

[14] Nascimento ABO, Balica HLL, Mayorga FDO, Silva MAM, Albuquerque IMAN, Almeida MB (2022). Limits and potentials of pediatric home care in a Ceará State municipality. Medicina (Ribeirao Preto).

[15] avalcante MEPL, Santos MM, Toso BRGO, Vaz EMC, Lima PMVM, Collet N (2022). It is better at home: characterization of home care services. Esc Anna Nery.

[16] Castro EAB, Leone DRR, Santos CM, Neta Gonçalves FCC, Gonçalves JRL, Contim D (2018). Home care organization with the Better at Home Program. Rev Gaucha Enferm.

[17] Lima ACB (2020). Análise da implantação do Serviço de Atendimento Domiciliar na óptica dos profissionais da Atenção Primária à Saúde.

[18] Maldonado TCP (2019). “Melhor em Casa!?” A resiliência do profissional frente à prática do atendimento domiciliar.

[19] Oliveira AV (2016). Análise do Programa Melhor em Casa: um olhar sobre a atenção domiciliar no âmbito do Sistema Único de Saúde (SUS).

[20] Canuto KF (2018). Melhor em Casa: um estudo de caso sobre a interdisciplinaridade no programa no município de Palmeira dos Índios/AL.

[21] Teixeira C, Rosa RG (2025). Home care after intensive care unit-discharge: global differences. Crit Care Sci.

[22] Gheno J, Weis AH (2021). Care transition in hospital discharge for adult patients: integrative literature review. Texto Contexto Enferm.

[23] Fittipaldi ALM, O’Dwyer G, Henriques P (2021). Health education in primary care: approaches and strategies envisaged in public health policies. Interface (Botucatu).

[24] Steindal SA, Nes AAG, Godskesen TE, Holmen H, Winger A, Österlind J (2023). Advantages and challenges of using telehealth for home-based palliative care: systematic mixed studies review. J Med Internet Res.

[25] Baptista JA, Camatta MW, Filippon PG, Schneider JF (2020). Singular therapeutic project in mental health: an integrative review. Rev Bras Enferm.

[26] Silva JL Teston EF, Marcon S Vieira VCL, Ferreira PC Andrade GKS (2022). Potentials and limits in home care shared between teams: a qualitative study. Rev Min Enferm.

[27] Ferraz EM, Mendonça FF, Carvalho BG, Nunes EFPA, Santini SML (2025). Permanent education in health: a strategy for strengthening the training and performance of municipal management teams in the Unified Health System. Physis.

[28] Nakata LC, Feltrin AFS, Chaves LDP, Ferreira JBB (2020). Concept of health care network and its key characteristics: a scoping review. Esc Anna Nery.

[29] Bezerra AM, El Akra KMA, Oliveira RMB, Marques FRB, Neves ET, Toso BRGO (2023). Children and adolescents with special healthcare needs: care in home care services. Esc Anna Nery.

[30] Silva KL, Braga PP, Silva AE, Lopes LFL, Souza TM (2022). Discourses on technologies in home care: contributions between innovating, inventing, and investing. Rev Gaucha Enferm.

[31] Silva AE, Duarte ED, Fernandes SJD (2022). Palliative care production for health professionals in the context of home care. Rev Bras Enferm.

[32] Ubessi LD, Meneses MN, Silva LDA, Coimbra VCC, Kantorski LP, Rocha CMF (2021). Permanent health education: experiencing ways to see, live, feel and build the Unified Health System. Saberes Plurais Educ Saude.

[33] Belga SMMF, Jorge AO, Silva KL (2022). Continuity of care from the hospital: interdisciplinarity and devices for integrality in health care networks. Saude Debate.

[34] Santos KM Tracera GMP, Nascimento FPB Moreira JPL, Ruas CAS Fonseca EC (2022). Work-related disorders and psychosocial risks in nursing professionals. Acta Paul Enferm.

