# Supplementary material for: Potential strengths and limitations of the Better at Home Program identified by professionals and managers: a scoping review of the literature
Source: Rev Lat Am Enfermagem. 2026 Jul 24;34:e4879. doi: 10.1590/1518-8345.8251.4879 (PMC13405591; doi:10.1590/1518-8345.8251.4879)
Supplement: Translation

# Translation: Potencialidades e fragilidades do Programa Melhor em Casa identificadas por profissionais e gestores: revisão de escopo^*^

**DOI:** 10.1590/1518-8345.8251.4880

**Authors:** Cristina Nunes Viana, Leticia Alves da Silva e Silva, Fernanda Cirne Lima Weston, Adriana Aparecida Paz

**Affiliations:** 1 Universidade Federal de Ciências da Saúde de Porto Alegre, Porto Alegre, RS, Brasil.; 2 Prefeitura Municipal de Porto Alegre, Secretaria Municipal de Saúde, Porto Alegre, RS, Brasil.; 3 Pontifícia Universidade Católica do Rio Grande do Sul, Porto Alegre, RS, Brasil.; 4 Novocure, DSS Learning and Operations Specialist, Porto Alegre, RS, Brasil.; 5 Bolsista da Coordenação de Aperfeiçoamento de Pessoal de Nível Superior (CAPES), Brasil.; 6 Bolsista do Conselho Nacional de Desenvolvimento Científico e Tecnológico (CNPq), Brasil.

**Keywords:** Serviços de Assistência Domiciliar, Serviços Hospitalares de Assistência Domiciliar, Assistência Domiciliar, Enfermagem Domiciliar, Pacientes Domiciliares, Educação Domiciliar.

## Abstract

**Objetivo:**

mapear na literatura científica as potencialidades e fragilidades do Programa Melhor em Casa identificadas por profissionais de saúde e gestores.

**Método:**

revisão de escopo embasada no manual do JBI e nas diretrizes PRISMA-ScR. A busca foi realizada nas bases PubMed/MEDLINE, LILACS, Scopus, Embase, *Web of Science*, CINAHL, e teses e dissertações CAPES, abrangendo publicações entre 2011 e 2024.

**Resultados:**

dos 552 estudos identificados, oito atenderam aos critérios de inclusão e foram analisados. As potencialidades do programa referem-se à promoção da desospitalização, fortalecimento do trabalho em rede, desenvolvimento de ações educativas em saúde com as famílias e flexibilidade na gestão do cuidado. Em contrapartida, foram evidenciadas fragilidades relacionadas à insuficiência da educação permanente dos profissionais, desgaste emocional das equipes, limitações tecnológicas, e dificuldades de interlocução com a Rede de Atenção à Saúde.

**Conclusão:**

o Programa Melhor em Casa é uma estratégia relevante no processo da desospitalização, contudo, enfrenta desafios estruturais e operacionais que comprometem sua sustentabilidade. Investimentos em suporte tecnológico e fortalecimento das equipes para melhorar a continuidade e a qualidade da atenção domiciliar são necessários. Registro *Open Science Framework*: DOI 10.17605/OSF.IO/6UCDB

## Destaques

**(1)** Fortalecimento do cuidado em rede favorece a desospitalização segura. **(2)** A educação em saúde promove autonomia de usuários e cuidadores. **(3)** O uso do teleatendimento amplia o acesso e qualifica o cuidado domiciliar. **(4)** Educação permanente em saúde é essencial para a efetividade do programa. **(5)** Falta de estrutura e apoio emocional fragilizam equipes da atenção domiciliar.

## Introdução

A atenção domiciliar (AD) constitui uma modalidade de cuidado prestado no domicílio do usuário, podendo ser realizada no âmbito da Atenção Primária à Saúde (APS) ou, em casos que demandam cuidados mais complexos, pelo Programa Melhor em Casa (PMeC). Nos últimos anos, a AD tem ganhado crescente relevância no Sistema Único de Saúde (SUS), especialmente por proporcionar um cuidado contínuo, humanizado e centrado nas necessidades dos usuários. Integrada à Rede de Atenção à Saúde (RAS), essa modalidade se destaca por oferecer assistência no ambiente domiciliar, priorizando a segurança e o bem-estar dos usuários^1^.

A AD promove ações de tratamento, reabilitação, paliação e prevenção, sendo indicada para usuários que estejam restritos ao leito ou ao domicílio, de forma temporária ou permanente, em razão de condições clínicas ou situações de vulnerabilidade^2^. Essa modalidade de cuidado reconhece o contexto social e cultural do usuário e de seus familiares, contribuindo para a redução do risco de infecções, da recorrência de hospitalizações e da utilização inadequada dos serviços hospitalares. Além disso, favorece a otimização na gestão eficiente dos leitos hospitalares e dos recursos em saúde disponíveis, servindo como uma “porta de saída” qualificada da rede de urgência e emergência, com potencial para diminuir a superlotação nesses serviços^3^.

O PMeC focaliza a desospitalização dos usuários que requerem acompanhamento clínico e suporte de uma equipe multiprofissional, promovendo não apenas o cuidado, mas incluindo as ações educativas aos usuários, familiares, responsáveis e cuidadores para a continuidade da assistência no domicílio. A atuação do PMeC ocorre por meio dos Serviços de Atenção Domiciliar (SAD), os quais são complementares à APS e aos serviços de urgência, podendo também ser substitutivos à hospitalização^2^.

Diante do envelhecimento populacional e da demanda crescente por uma atenção à saúde mais integral e humanizada, os serviços de AD têm se expandido em todo o mundo^4^. No contexto brasileiro, os SAD começaram a se estruturar com maior intensidade a partir de 1990, sendo incorporados de forma estratégica e definitiva ao SUS em 2011, com a criação do PMeC^5^.

Fatores como a transição demográfica e epidemiológica acelerada, o aumento dos custos em saúde, o processo de desinstitucionalização e a busca por modelos mais humanizados de cuidado evidenciam a importância da expansão do PMeC no cenário nacional^3^ ^-^ ^5^. Para aprimorar sua gestão, torna-se importante compreender as percepções dos profissionais e gestores sobre o programa quanto aos desafios e potencialidades.

As equipes multiprofissionais que atuam no PMeC vivenciam, na prática da assistência prestada aos usuários, diferentes barreiras e desafios, sendo, portanto, uma fonte valiosa para a identificação de elementos que possam subsidiar melhorias na condução do programa. No entanto, apesar da relevância de suas experiências, não foram identificadas, na literatura científica, revisões que reunissem as percepções dos profissionais e gestores sobre o PMeC, desde sua criação. Nesse contexto, esta revisão teve o objetivo de mapear na literatura científica as potencialidades e fragilidades do Programa Melhor em Casa identificadas por profissionais de saúde e gestores.

## Método

### Tipo de estudo

Trata-se de uma revisão de literatura baseada em evidências científicas do tipo revisão de escopo, guiada pelas recomendações do JBI *Reviewer’s Manual*, o qual orienta o mapeamento de conceitos-chave, a clarificação de áreas de pesquisa e a identificação de lacunas do conhecimento^6^. Para o desenvolvimento do estudo foram percorridas cinco etapas propostas pelo JBI: 1) Formulação da questão de pesquisa; 2) Identificação dos estudos relevantes; 3) Seleção dos estudos; 4) Extração dos dados; e 5) Síntese e apresentação dos resultados. O protocolo de pesquisa foi registrado na plataforma *Open Science Framework* sob o DOI 10.17605/OSF.IO/6UCDB ^7^. A extensão *Preferred Reporting Items for Systematic Reviews and Meta-Analyses for Scoping Reviews* (PRISMA-ScR) foi utilizada para reportar com transparência na condução dos resultados da análise de escopo^8^. O estudo foi realizado na cidade de Porto Alegre, no estado do Rio Grande do Sul, Brasil, no período de fevereiro de 2024 a março de 2025.

### Critérios de seleção

Foram considerados como critérios de inclusão: estudos publicados na íntegra, disponíveis eletronicamente, sem restrição de idioma, que respondessem à questão de pesquisa proposta. Estabeleceu-se como recorte temporal o período de 2011 a 2024, em razão da homologação da Portaria do PMeC pelo Ministério da Saúde (MS) em 2011^9^, abrangendo assim treze anos de implementação do programa no território brasileiro. Foram excluídos editoriais, cartas ao editor, sites, notícias e resumos de eventos científicos.

### Coleta de dados

Para a estratégia de busca da revisão foi adotada a estrutura mnemônica PCC, considerando: População (P) - profissionais de saúde e gestores; Conceito (C) - potencialidades e fragilidades do PMeC; e Contexto (C) - Brasil. A questão formulada foi: “*Quais são as potencialidades e fragilidades observadas pelos profissionais de saúde e gestores sobre o Programa Melhor em Casa no Brasil?”*

Na estratégia de busca foram incluídos termos presentes na estrutura PCC, utilizou-se descritores identificados nos tesauros da área da saúde como *Medical Subject Headings* (MeSH) e Descritores em Ciências da Saúde (DeCS). Sendo um programa exclusivo do contexto brasileiro, a pesquisa contou com o apoio de uma bibliotecária para a construção da estratégia de busca, sendo sugerido utilizar o próprio nome do programa em português “Programa Melhor em Casa” e, em inglês, “*Better at home*”, a fim de ter uma maior cobertura de bibliografia acerca da temática e ampliar a estratégia de busca (Figura 1).

A busca de evidências científicas foi realizada nas seguintes fontes de dados: Literatura Latino-Americana e do Caribe em Ciências da Saúde (LILACS); *Public Medical Literature Analysis and Retrievel System Online* (PubMed/MEDLINE); Scopus; *Excerpta Medica data BASE* (EMBASE); *Web of Science* (WoS); e *Cumulative Index to Nursing and Allied Health Literature* (CINAHL). A literatura cinzenta foi pesquisada na Biblioteca Digital Brasileira de Teses e Dissertações da CAPES.

**Figura 1:** Estratégias de busca e bases de dados utilizadas na revisão de literatura. Porto Alegre, RS, Brasil, 2025

Base de dados	Estratégia de busca
LILACS*	(“melhor em casa” OR “programa melhor em casa”) AND programa AND (year_cluster:[2011 TO 2024]) AND ( db:(“LILACS”)) AND (year_cluster:[2011 TO 2024])
PubMed/MEDLINE	(“Better at home” OR “Better at home program”) AND program AND (brazil OR brazilian) Filters: from 2011 - 2024 (((“better”[All Fields] AND (“home environment”[MeSH Terms] OR (“home”[All Fields] AND “environment”[All Fields]) OR “home environment”[All Fields] OR “home”[All Fields])) OR (“better”[All Fields] AND (“home environment”[MeSH Terms] OR (“home”[All Fields] AND “environment”[All Fields]) OR “home environment”[All Fields] OR “programmable”[All Fields] OR “programmably”[All Fields] OR “programme”[All Fields] OR “programme s”[All Fields] OR “programmed”[All Fields] OR “programmer”[All Fields] OR “programmer s”[All Fields] OR “programmers”[All Fields] OR “programmes”[All Fields] OR “programming”[All Fields] OR “programmings”[All Fields] OR “programs”[All Fields]))) AND (“program”[All Fields] OR “program s”[All Fields] OR “programe”[All Fields] OR “programed”[All Fields] OR “programes”[All Fields] OR “programing”[All Fields] OR “programmability”[All Fields] OR “programmable”[All Fields] OR “programmably”[All Fields] OR “programme”[All Fields] OR “programmes”[All Fields] OR “programmed”[All Fields] OR “programmer”[All Fields] OR “programmers”[All Fields] OR “programmers”[All Fields] OR “programmes”[All Fields] OR “programming”[All Fields] OR “programmings”[All Fields] OR “programs”[All Fields]) AND (“brazil”[MeSH Terms] OR “brazil”[All Fields] OR “brazil”[All Fields] OR”home”[All Fields]) AND (“program”[All Fields] OR “programs”[All Fields] OR “programe”[All Fields] OR “programed”[All Fields] OR “programes”[All Fields] OR “programing”[All Fields] OR “programmability”[All Fields] OR “brazil”[All Fields] OR (“brazilian people”[Supplementary Concept] OR “brazilian people”[All Fields] OR “brazilians”[All Fields] OR “brazilian”[All Fields]))) AND (2011:2024[pdat])
Scopus	TITLE-ABS-KEY ( ( “Better at home” OR “Better at home program” ) AND program ) AND PUBYEAR > 2010 AND PUBYEAR < 2024
EMBASE	(‘better at home’ OR ‘better at home program’) AND ‘program’ AND [2011-2024]/py
WoS^†^	(“Better at home” OR “Better at home program”) AND program (All Fields) and 2018 or 2019 or 2023 or 2022 (Publication Years)
CINAHL	(‘better at home’ OR ‘better at home program’) AND ‘program’
CAPES Catálogo de Teses e Dissertações	“melhor em casa”

*LILACS = Literatura Latino-Americana e do Caribe em Ciências da Saúde; ^†^WoS = *Web of Science*

### Seleção de estudos

Todos os registros recuperados nas bases de dados foram identificados, agrupados e carregados no aplicativo Rayyan^®^ (*Intelligent Systematic Review*), onde foram removidas as duplicatas e realizada a triagem inicial dos títulos e resumos^10^. A literatura cinzenta foi analisada em planilhas do Excel^®^, também por meio de leitura inicial de título e resumo. Após a retirada das duplicatas no Rayyan^®^, procedeu-se à etapa inicial de análise dos dados, as revisoras realizaram a leitura independente e cega de títulos e resumos de todos os documentos na plataforma Rayyan^®^ e na planilha do Excel^®^. As revisoras atribuíram conceitos de aceitação ou rejeição, de acordo com a questão de pesquisa da revisão. O cegamento da plataforma Rayyan^®^ foi aberto em reunião entre as revisoras, a fim de verificar as divergências. Nesse sentido, os estudos que permaneciam com divergências foram avaliados por uma terceira revisora, que fez a avaliação na plataforma Rayyan^®^. Quanto à literatura cinzenta, um encontro presencial foi realizado com as duas revisoras, a fim de avaliar os resultados e discutir as divergências, de modo que não foi necessária a intervenção de uma terceira revisora.

Os estudos selecionados, foram lidos na íntegra e analisados descritivamente em conformidade com a questão de pesquisa e com a relevância do objetivo do estudo. No que tange à literatura cinzenta, uma dissertação em específico não foi encontrada na íntegra, tentou-se contato com o autor e orientadora por meio do correio eletrônico sem sucesso, e a outra dissertação foi fruto de um artigo selecionado no estudo, assim, optou-se pela utilização do artigo publicado.

### Tratamento dos dados

Com base nos documentos selecionados, os dados extraídos foram organizados em uma planilha do Excel^®^, contendo informações sobre: autor, título, tipo de estudo, ano de publicação, idioma, amostra, periódico de publicação, país de origem, ano da publicação, objetivos do estudo, principais resultados e conclusões. A análise dos dados ocorreu de forma descritiva e, posteriormente, os achados foram categorizados em potencialidades e fragilidades do PMeC por meio de uma análise temática.

### Aspectos éticos

Por se tratar de uma revisão de escopo, não houve necessidade de submissão do estudo ao Comitê de Ética em Pesquisa. Afirma-se que não houve conflito de interesses por parte das pesquisadoras envolvidas e que foram respeitados os direitos autorais dos documentos citados conforme a Lei nº 12.853 de 2013^11^.

## Resultados

Na busca pelas bases de dados, identificou-se 552 publicações. Após a remoção de 45 duplicatas, restaram 507 estudos. Em seguida, procedeu-se à leitura dos títulos e resumos, aplicando-se os critérios de inclusão e exclusão por dois revisores independentes, resultando na seleção de 27 estudos. Posteriormente, fez-se a leitura na íntegra dos documentos selecionados, considerando os critérios de inclusão e exclusão estabelecidos, culminando em uma amostra final constituída por oito publicações (Figura 2).

**Figura 2:** Fluxograma PRISMA- ScR^12^ utilizado para identificação e seleção de estudos. Porto Alegre, RS, Brasil, 2025

O principal idioma das publicações foi o português (n=5; 62,5%), seguido do idioma inglês (n=3; 37,5%). Quanto ao delineamento metodológico utilizado pelos autores, prevaleceram os estudos de abordagem qualitativa (n=6; = 75%), seguidos por estudos quantitativos (n=1;12,5%) e métodos mistos (n=1; 12,5%).

Nos estudos, identificou-se 90 participantes. Em relação ao ano de publicação, identificou-se que os artigos foram publicados entre 2016 a 2022, sendo o maior número no ano de 2022 (n=3; 37,5%), seguido pelos anos de 2018 (n=2; 25%), 2020 (n=1;12,5%), 2019 (n=1;12,5%), e 2016 (n=1; 12,5%). A Figura 3 apresenta a caracterização dos estudos incluídos na revisão, com informações sobre autoria, título, tipo de estudo, ano de publicação e número de participantes.

**Figura 3:** Caracterização dos estudos identificados na revisão. Porto Alegre, RS, Brasil, 2025

Estudo	Autor	Título	Tipo de estudo	Ano de publicação	Amostra (n=)
E1^13^	Verdi DS, Pires RRC	*Between standardization and flexibility: the implementation of federal program Better at Home considering local diversity*	Estudo descritivo com abordagem qualitativa	2022	6
E2^14^	Almeida MB, Albuquerque IMAN, Silva MAM, Mayorga FDO, Balica HLL, Nascimento ABO	*Limits and Potentials of Pediatric Home Care in a Ceará State municipality*	Estudo avaliativo com abordagem qualitativa	2022	-
E3^15^	Cavalcante MEPL, Santos MM, Toso BRGO, Vaz EMC, Lima PMVM, Collet N	*It is better at home: characterization of home care services*	Estudo exploratório quantitativo	2022	23
E4^16^	Castro EAB, Leone DRR, Santos CM, Neta FCCG, Gonçalves JRL, Contim D, Silva KL	*Home care organization with the Better at Home Program*	Estudo descritivo, exploratório com abordagem qualitativa	2018	18
E5^17^	Lima ACB	Análise da implantação do serviço de atendimento domiciliar na óptica dos profissionais da atenção primária à saúde	Estudo descritivo, exploratório com abordagem qualitativa	2020	14
E6^18^	Maldonado TCP	“Melhor em Casa!?” A resiliência do profissional frente a prática do atendimento domiciliar	Estudo de casos múltiplos com abordagem qualitativa	2019	9
E7^19^	Oliveira AV Neto	Análise do Programa Melhor em Casa: um olhar sobre a atenção domiciliar no âmbito do Sistema Único de Saúde	Estudo descritivo, exploratório quantitativo e abordagem qualitativa	2016	6
E8^20^	Canuto KF	Melhor em Casa: um estudo de caso sobre a interdisciplinaridade no programa no município de Palmeira dos Índios, Alagoas	Estudo avaliativo com abordagem qualitativa	2018	14

A Figura 4 sintetiza os objetivos dos estudos e os principais resultados de cada estudo, permitindo visualizar as percepções dos profissionais acerca do PMeC.

**Figura 4:** Síntese descritiva dos estudos incluídos na revisão de escopo. Porto Alegre, RS, Brasil, 2025

Número	Objetivos	Principais percepções dos profissionais
E1^13^	Conhecer as diferentes experiências de gestores locais com os direcionamentos e normatizações federais relativos à implementação do Programa Melhor em Casa.	Como potencialidades, observa-se a portaria norteadora do programa como adequada à realidade. Percebe-se a importância de instrumentos de coordenação federal com adaptações locais, e a necessidade de flexibilização nas recomendações da portaria do PMeC*. A adesão ao programa trouxe maiores recursos financeiros à AD^†^, adequando a equipe de saúde e o fluxo assistencial. O PMeC* permitiu que óbitos ocorram no domicílio e proporciona a desospitalização. No entanto, ainda se observa resistência por parte da equipe médica hospitalar em encaminhar pacientes ao programa, além de dificuldades na composição das equipes de AD^†^.
E2^14^	Conhecer os limites e potencialidades da assistência domiciliar pediátrica do Programa Melhor em Casa em um município do estado do Ceará.	A falta de conhecimento dos profissionais de diferentes níveis assistenciais sobre a AD^†^ prejudica a continuidade do cuidado, bem como é citada a ausência de algumas categorias profissionais, falta de estrutura física exclusiva para o PMeC* e deficiência de insumos essenciais para os cuidados. Identificou-se a carência em transporte, falta de capacitações, falta de laços entre o programa e gestão da APS^‡^. Outra característica negativa citada foi a mudança de profissionais a cada ciclo de gestão, cortando o vínculo entre os profissionais, pacientes e a rede. Todavia, como um critério de melhoria está a redução de internações hospitalares pediátricas.
E3^15^	Caracterizar os Serviços de Atenção Domiciliar em funcionamento na Paraíba.	O uso do teleatendimento apresenta-se como grande estratégia de cuidado aos usuários. Como pontos que podem melhorar, encontra-se o transporte do SAD^§^ ao qual é compartilhado com demais serviços da rede. E algumas equipes não cumprem carga horária mínima exigida, atuam em alguns turnos, refletindo na continuidade da assistência. As sedes do SAD^§^ geralmente estão em anexo a outros serviços de saúde, mesmo com a interligação, ocorre fragmentação na comunicação entre os serviços. No que tange ao prontuário eletrônico do paciente e PTS^||^, são realizados em apenas 50% dos serviços, mostrando ainda a fragmentação em alguns pontos essenciais, para o conhecimento das informações dos pacientes e planejamento do cuidado individualizado.
E4^16^	Compreender a organização da Atenção Domiciliar no contexto da atenção à saúde pública ofertada por municípios que aderiram ao Programa Melhor em Casa no Estado de Minas Gerais.	Percebem a integração com a rede como uma fragilidade do PMeC*. Realizar reuniões entre equipes de diferentes serviços, e a organização de processos são vistos como estratégias eficazes para a melhoria do atendimento ao usuário na AD^†^.
E5^17^	Analisar como ocorreu o processo de implantação do Serviço de Atenção Domiciliar na perspectiva dos profissionais da atenção primária à saúde do município de Araranguá/Santa Catarina.	Evidenciado a importância do PMeC* na desospitalização de pacientes, na melhoria da qualidade de vida dos usuários e familiares, sendo uma referência para a APS^‡^ e hospitalar. Como pontos de melhoria, apresenta-se a falta de compartilhamento de cuidados, a dificuldade de interação entre profissionais e o desconhecimento de encaminhamentos pela APS^‡^. Ainda, há sobrecarga da equipe SAD^§^, falta de estrutura física e transporte, dificuldade da responsabilização do usuário pelo cuidado. Apresenta-se como sugestão a educação permanente em saúde com os profissionais da RAS^¶^.
E6^18^	Compreender a experiência de profissionais do Programa Melhor em Casa à luz da resiliência.	O PMeC* é visto como um serviço acolhedor, resolutivo, que realiza um trabalho multiprofissional humanizado, com um olhar além das demandas clínicas. O atendimento abrange as famílias com suporte e acolhimento, tornando-se um serviço diferenciado na RAS^¶^. O serviço adentra nos lares e, por ter um contato tão íntimo com situações diversas, os profissionais estão expostos à sobrecarga, impotência e fragilidade. Devido ao grande vínculo com as famílias, algumas apresentam receio em receber alta do PMeC*.
E7^19^	Caracterizar a implantação do Programa Melhor em Casa e o analisar sob a perspectiva dos gestores municipais.	O PMeC* é percebido como uma alternativa à internação hospitalar, apresenta grande potencial de desospitalização e articulação com a RAS^¶^. Otimiza leitos hospitalares, adentra na rede para discussão de casos e compartilhar cuidados. Deve ser dada uma maior atenção à divulgação do programa pelo Ministério da Saúde à população, sendo necessário maior explanação da portaria com a RAS^¶^, a fim de melhor entendimento. Incentivos para incremento da qualidade técnica da equipe do PMeC* e correto fornecimento de insumos e capacitações a APS^‡^ para encaminhamento adequado de pacientes.
E8^20^	Avaliar a compreensão dos profissionais sobre a interdisciplinaridade nas ações do Programa Melhor em Casa no Município de Palmeira dos Índios/Alagoas.	A interdisciplinaridade ainda tem seu significado pouco conhecido, ou definido de forma equivocada entre os profissionais. No SAD^§^, ela ocorre por meio de ações no dia a dia do serviço, em relações horizontais com trocas ou atendimento compartilhado. O trabalho em equipe está intimamente ligado, a falta de veículos exclusivos ao SAD^§^, ou o uso de um veículo exclusivo para curativos, mostra a falta de compartilhamento do cuidado. As admissões são realizadas apenas por um profissional do serviço social, onde não são realizadas trocas, não sendo realizado o PTS^||^. Ocorre a necessidade de melhorias no relacionamento interpessoal, encontros de equipe, atendimento multiprofissional, bem como a divulgação dos critérios do programa para a RAS^¶^.

*PMeC = Programa Melhor em Casa; ^†^AD = Atendimento Domiciliar; ^‡^APS = Atenção Primária à Saúde; ^§^SAD = Serviço de Atendimento Domiciliar; ^||^PTS = Plano Terapêutico Singular; ^¶^RAS = Rede de Atenção à Saúde

Para favorecer a compreensão e organização dos resultados, bem como responder à questão de pesquisa da revisão, os achados foram subdivididos em duas categorias temáticas: Potencialidades e Fragilidades do PMeC, conforme Figura 5.

**Figura 5:** Categorias temáticas da revisão de escopo. Porto Alegre, RS, Brasil, 2025

Categorias Temáticas
Potencialidades do Programa Melhor em Casa
Construção do trabalho em rede^13^ ^-^ ^14^ ^),(^ ^16^ ^),(^ ^18^ ^-^ ^20^; Integração do serviço com a sociedade^13^; Protocolos e processos organizacionais^16^; Portaria norteadora adequada à realidade^13^; Desospitalização e redução de internações desnecessárias^13^ ^-^ ^14^ ^),(^ ^17^; Educação em saúde com famílias^14^ ^),(^ ^16^ ^-^ ^17^; Teleatendimento^15^; Plano Terapêutico Singular (PTS)^16^; Flexibilidade e autonomia por parte da gestão^20^; Humanização^15^; e Melhora na qualidade de vida dos usuários^17^.
**Fragilidades do Programa Melhor em Casa**
Educação permanente^14^ ^-^ ^20^; Interlocução com a RAS*^13^ ^-^ ^19^; Estruturação dos serviços^13^ ^-^ ^17^ ^),(^ ^20^; Responsabilização do usuário pelo cuidado^19^ ^-^ ^20^; Desgaste emocional dos profissionais^18^ ^-^ ^19^; Falta de recursos tecnológicos^15^; Uso do prontuário eletrônico^15^; e Fragmentação da equipe^20^.

*RAS = Rede de Atenção à Saúde

## Discussão

Os achados desta revisão de escopo evidenciaram duas categorias temáticas que caracterizam a atuação do programa no cenário brasileiro: potencialidades e as fragilidades do PMeC. A análise crítica permite compreender os avanços alcançados e os desafios persistentes na consolidação da AD como estratégia integrada à RAS.

### Potencialidades para o Programa Melhor em Casa

A construção do trabalho em rede, enquanto eixo estruturante do PMeC, destacou-se como a principal potencialidade evidenciada nos estudos analisados. A atuação articulada entre os distintos pontos da RAS mostra-se fundamental, considerando que o programa assiste, majoritariamente, usuários procedentes de internações hospitalares recentes. Tal configuração demanda a articulação com a rede intra-hospitalar para garantir a transição segura do cuidado, o estabelecimento de vínculo intrafamiliar da equipe do PMeC no domicílio e, posteriormente, a integração do usuário na APS, compondo um contínuo assistencial na RAS. Nesse contexto, a consolidação do trabalho em rede se faz necessário e está intrinsecamente relacionada à estrutura do serviço, contribuindo para obtenção de melhores desfechos clínicos. Uma revisão narrativa destacou que a qualidade do atendimento domiciliar está relacionada tanto pela capacidade dos profissionais em acolher as necessidades quanto pelo estabelecimento de uma comunicação direta com a família^21^. Isso reforça a articulação com os diferentes níveis da RAS, o que favorece a formulação de estratégias para ações integradas que atendam, de maneira multidisciplinar, às necessidades e expectativas do usuário e família diante das incertezas associadas ao cuidado domiciliar.

Outro ponto evidenciado nos estudos refere-se à desospitalização e à redução de internações consideradas evitáveis, corroborando com os objetivos centrais do programa, que visam à transição segura de usuários estáveis para o domicílio. O retorno do usuário ao ambiente domiciliar configura-se como uma ação de continuidade do cuidado iniciado no contexto hospitalar e, quando realizado de forma planejada e com acesso aos recursos necessários, permite a manutenção da articulação com os diferentes níveis da rede, de maneira integrada, resolutiva e centrada na pessoa. A programação de uma alta segura implica na avaliação das necessidades clínicas e sociais do usuário, bem como na estrutura familiar e na disponibilidade de serviços da RAS, elementos fundamentais para a prevenção de reinternações^22^. Ainda nesse contexto, a redução das internações desnecessárias é favorecida pelo reconhecimento precoce de alterações no quadro clínico do usuário e pela adequada condução na atenção às intercorrências, sendo isso resultado do processo educativo promovido pelas equipes junto às famílias, o que contribui para a diminuição do retorno do usuário ao ambiente hospitalar.

A educação em saúde dirigida às famílias foi representada nos estudos como uma das estratégias diferenciais da atuação do PMeC. O programa ultrapassa o enfoque na desospitalização ao investir em ações educativas com cuidadores e familiares no espaço domiciliar, ambiente no qual ocorrem intensas trocas de saberes. Nesse contexto íntimo e singular, marcado pela reorganização da rotina familiar diante da presença de familiar adoecido, é essencial que a equipe mostre empatia e comprometimento para, progressivamente, promover a construção de conhecimentos entre os familiares para o exercício do cuidado seguro e integral. A educação em saúde deve, portanto, ser concebida como uma ferramenta para a promoção da autonomia e do protagonismo de todos os envolvidos, incentivando a corresponsabilização no processo de cuidado^23^.

A adoção do teleatendimento e do PTS também foi destacada como estratégia que personaliza o cuidado, colocando o usuário como agente central do seu processo de reabilitação e promovendo maior agilidade e efetividade nas ações assistenciais. O teleatendimento constitui-se em um sistema de apoio que permite ao usuário permanecer em casa, assim como os recursos visuais da plataforma possibilitam o desenvolvimento de relações interpessoais com os profissionais de saúde ao longo do tempo. A modalidade é uma alternativa viável para monitorar sintomas, orientar ações e incentivar à adesão ao cuidado domiciliar, explorando as possibilidades tanto assistenciais quanto educativas^24^. No que se refere ao planejamento do cuidado, o PTS apresenta-se como um instrumento que serve de guia para as ações da equipe multiprofissional, sendo compartilhado entre os membros da equipe, com vistas à implementação de intervenções alinhadas às necessidades singulares de cada usuário^25^ ^-^ ^26^.

### Fragilidades para o Programa Melhor em Casa

As fragilidades mais recorrentes identificadas nos estudos foram a educação permanente e a interlocução com a RAS, evidenciando dois aspectos centrais que demandam atenção qualificada no contexto nacional. Outro aspecto frequentemente apontado nos estudos refere-se à estrutura dos serviços, seguido da responsabilização do usuário pelo cuidado e pelo desgaste emocional dos profissionais envolvidos na AD.

A educação permanente, reconhecida como uma estratégia de ensino-aprendizagem em serviço, com princípios problematizadores, interdisciplinares e interprofissionais em um contexto real, tem o foco nas necessidades cotidianas da prática profissional para a qualificação da assistência prestada ao usuário, sobretudo no PMeC, dada a complexidade do cuidado domiciliar. Esse processo de educação tem proporcionado transformações graduais nas práticas na saúde, com o intuito ao alcance de melhoria do cuidado, da reorganização do processo de trabalho e da qualidade da gestão pública, por meio de ações pedagógicas inovadoras e alinhadas ao modelo assistencial vigente^27^. Tais ações devem contemplar metodologias inovadoras, incluindo aprendizagem virtual e recursos tecnológicos, que favoreçam o desenvolvimento de competências dos trabalhadores da saúde^4^.

A fragmentação na interlocução com a RAS ou as dificuldades de acesso em situações de intercorrência com os usuários no PMeC configuram obstáculos relevantes que levam ao desgaste emocional nas equipes multiprofissionais, assim como à resolutividade das necessidades do usuário. Essas barreiras comprometem a integralidade da atenção, podendo ocasionar descontinuidade do cuidado, reincidência de agravos de saúde e reinternações evitáveis, frequentemente relacionadas à fragilidade da articulação entre os serviços que compõem a rede. A integração efetiva entre os serviços da RAS, com base em uma visão compartilhada, adoção de diretrizes clínicas e organização horizontal, é imperativa para a qualificação do cuidado e redução de redundâncias^28^.

A estruturação dos serviços, por sua vez, abrange desde limitações nas instalações físicas inadequadas até a insuficiência de veículos, frequentemente compartilhados com outros serviços ou unidades do município, comprometendo a realização das visitas domiciliares. A oferta adequada de recursos materiais, embora essencial, não é suficiente para assegurar a efetividade da assistência no contexto domiciliar. Para que a AD se realize de maneira eficiente e segura, faz-se necessário um planejamento estratégico que incorpore a educação permanente e garanta a alocação adequada de recursos materiais e de uma qualificada gestão de pessoas^28^.

Outro aspecto emergente nos estudos refere-se à necessidade de incorporação de novas tecnologias, como o PEP. A ausência desse recurso limita o compartilhamento de informações entre os serviços da RAS, dificultando a continuidade e integralidade do cuidado. A implementação do PEP, além de proporcionar maior praticidade e agilidade na gestão das informações, é essencial para a expansão da AD^29^. O PEP contribui para a segurança e eficácia do atendimento, permitindo o acesso ao histórico do usuário e subsidiando a tomada de decisão clínica^26^.

A responsabilização do usuário e da família pelo cuidado é um desafio em alguns estudos. Tal aspecto requer abordagem desde a admissão do usuário no programa, por meio de pactuações claras com a família quanto aos processos de cuidado e à alta, assegurando que usuários e cuidadores estejam instrumentalizados para o cuidado, de modo que desperte um interesse, corresponsabilidade e compartilhamento de saberes e conhecimento^30^. É necessário promover a autonomia da família no processo de corresponsabilização incentivando o protagonismo dos envolvidos, e consolidar a AD como uma referência para o cuidado sempre que necessário^31^. A AD configura-se como uma nova alternativa inovadora de produção do cuidado fora das instituições tradicionais, como hospitais, permitindo que a família e o cuidador assumam papel central na gestão do cuidado no domicílio, contribuindo para a construção de estratégias mais adequadas às realidades do território^32^.

Outro aspecto de vulnerabilidade identificado em dois estudos foi o desgaste emocional dos profissionais, especialmente no cuidado aos usuários com doenças crônicas. A atuação no domicílio expõe o profissional a contextos adversos, incluindo condições precárias de habitação e escassez de recursos, condições que potencializam o sofrimento psíquico e a sobrecarga emocional. Tais condições exigem a atenção dos gestores quanto à saúde mental das equipes, por meio de ações de educação em saúde, espaços de escuta e apoio psicossocial^33^. A exposição contínua a riscos psicossociais pode resultar em repercussões físicas, mentais e sociais significativas, frequentemente negligenciados por preconceitos que relacionam o sofrimento psíquico à fragilidade individual, em detrimento da compreensão das condições laborais envolvidas no cotidiano na AD^34^.

Entre as limitações do estudo, destaca-se a escassa disponibilidade de produções científicas sobre a temática, o que evidencia a necessidade de investigações adicionais que permitam conhecer de forma mais abrangente o potencial do programa e identificar melhorias que possam ser aplicadas ao PMeC. Ao considerar as diversidades culturais e geográficas do país, muitas ações podem ser adaptadas, recriadas e expandidas. A educação permanente também se apresenta como uma limitação relevante, indicando a pertinência de desenvolver estudos de intervenção voltados à qualificação dos profissionais no PMeC.

As contribuições deste estudo para a enfermagem são significativas, ao oferecer uma análise crítica e abrangente do PMeC, destacando suas potencialidades e fragilidades no contexto da AD integrada à RAS. A atuação da enfermagem no contexto da equipe multiprofissional no PMeC revela-se essencial na articulação do cuidado em rede, na educação em saúde às famílias e na promoção da continuidade assistencial segura e centrada no usuário. O estudo fornece subsídios para o aprimoramento das práticas profissionais e contribui para o fortalecimento da AD como estratégia inovadora e resolutiva no que tange à educação permanente, uso de tecnologias como o teleatendimento e o prontuário eletrônico, e a valorização do cuidado à saúde mental diante do desgaste emocional das equipes. Dessa forma, reforça-se o papel estratégico da enfermagem na coordenação do cuidado, na qualificação da atenção prestada no domicílio e na construção de modelos assistenciais mais sustentáveis, eficientes e humanizados.

## Conclusão

O mapeamento das produções científicas sobre as potencialidades e fragilidades do PMeC, identificadas por profissionais de saúde e gestores, mostrou-se limitado em quantidade quando comparado a outras áreas de conhecimento na saúde. Como potencialidades do PMeC, identificou-se a construção do trabalho em rede, a desospitalização, a redução de reinternações evitáveis, a educação em saúde junto às famílias, o teleatendimento, a utilização do PTS, a flexibilidade e autonomia na gestão, bem como a humanização e a melhoria da qualidade de vida dos usuários. No que se refere às fragilidades observadas, destacam-se aspectos que demandam medidas qualificadas para o aprimoramento do programa e da atenção à saúde no âmbito domiciliar: a educação permanente, a interlocução com a RAS, a estrutura dos serviços, a corresponsabilização do usuário e familiares pelo cuidado, o desgaste emocional dos profissionais, a insuficiência de recursos tecnológicos, a ausência do PEP e a fragmentação da equipe.

Dessa forma, o estudo evidencia a relevância do PMeC no sistema de saúde brasileiro, ao mostrar seu potencial para qualificar o cuidado domiciliar. Para isso, são necessárias intervenções estratégicas e investimentos estruturais que fortaleçam o programa e assegurem sua efetividade e sustentabilidade a longo prazo. Recomenda-se, ainda, a realização de novos estudos que aprofundem os temas abordados, contribuindo para o avanço do conhecimento e o aprimoramento das práticas no âmbito da AD oferecida pelo PMeC.

## Data Availability

Todos os dados gerados ou analisados durante este estudo estão incluídos neste artigo publicado.

# Translation: **Potencialidades y fragilidades del Programa *Melhor em Casa* identificadas por profesionales y gestores: revisión de alcance** ^*^

**DOI:** 10.1590/1518-8345.8251.4878

**Authors:** Cristina Nunes Viana, Leticia Alves da Silva e Silva, Fernanda Cirne Lima Weston, Adriana Aparecida Paz

**Affiliations:** 1 Universidade Federal de Ciências da Saúde de Porto Alegre, Porto Alegre, RS, Brasil.; 2 Prefeitura Municipal de Porto Alegre, Secretaria Municipal de Saúde, Porto Alegre, RS, Brasil.; 3 Pontifícia Universidade Católica do Rio Grande do Sul, Porto Alegre, RS, Brasil.; 4 Novocure, DSS Learning and Operations Specialist, Porto Alegre, RS, Brasil.; 5 Bolsista da Coordenação de Aperfeiçoamento de Pessoal de Nível Superior (CAPES), Brasil.; 6 Bolsista do Conselho Nacional de Desenvolvimento Científico e Tecnológico (CNPq), Brasil.

**Keywords:** Servicios de Atención de Salud a Domicilio, Servicios de Hospitalización a Domicilio Provisto por Hospital, Atención Domiciliaria de Salud, Cuidados de Enfermería en el Hogar, Personas Imposibilitadas, Educación en Casa.

## Abstract

**Objetivo:**

mapear en la literatura científica las potencialidades y fragilidades del Programa *Melhor em Casa* identificadas por profesionales de la salud y gestores.

**Método:**

revisión de alcance basada en el manual del JBI y en las directrices PRISMA-ScR. La búsqueda se realizó en las bases PubMed/MEDLINE, LILACS, Scopus, Embase, *Web of Science*, CINAHL, y en tesis y disertaciones CAPES, abarcando publicaciones entre 2011 y 2024.

**Resultados:**

de los 552 estudios identificados, ocho cumplieron los criterios de inclusión y fueron analizados. Las potencialidades del programa se refieren a la promoción de la deshospitalización, el fortalecimiento del trabajo en red, el desarrollo de acciones educativas en salud con las familias y la flexibilidad en la gestión del cuidado. En contrapartida, se evidenciaron fragilidades relacionadas con la insuficiencia de la educación permanente de los profesionales, el desgaste emocional de los equipos, las limitaciones tecnológicas y las dificultades de interlocución con la Red de Atención a la Salud.

**Conclusión:**

el Programa *Melhor em Casa* es una estrategia relevante en el proceso de deshospitalización; sin embargo, enfrenta desafíos estructurales y operativos que comprometen su sostenibilidad. Se requieren inversiones en soporte tecnológico y fortalecimiento de los equipos para mejorar la continuidad y la calidad de la atención domiciliaria. Registro *Open Science Framework*: DOI 10.17605/OSF.IO/6UCDB

## Destacados

**(1)** El fortalecimiento de la atención en red favorece la deshospitalización segura. **(2)** La educación en salud promueve la autonomía de usuarios y cuidadores. **(3)** El uso de la teleatención amplía el acceso y cualifica la atención domiciliaria. **(4)** La educación permanente en salud es esencial para la efectividad del programa. **(5)** La falta de estructura y de apoyo emocional fragiliza a los equipos de atención domiciliaria.

## Introducción

La atención domiciliaria (AD) constituye una modalidad de cuidado prestado en el domicilio del usuario, pudiendo realizarse en el ámbito de la Atención Primaria de Salud (APS) o, en casos que demandan cuidados más complejos, por el Programa *Melhor em Casa* (PMeC). En los últimos años, la AD ha adquirido creciente relevancia en el Sistema Único de Salud (SUS), especialmente por proporcionar un cuidado continuo, humanizado y centrado en las necesidades de los usuarios. Integrada a la Red de Atención a la Salud (RAS), esta modalidad se destaca por ofrecer asistencia en el entorno domiciliario, priorizando la seguridad y el bienestar de los usuarios^1^.

La AD promueve acciones de tratamiento, rehabilitación, cuidados paliativos y prevención, y está indicada para usuarios que estén restringidos al lecho o al domicilio, de forma temporal o permanente, debido a condiciones clínicas o situaciones de vulnerabilidad^2^. Esta modalidad de cuidado reconoce el contexto social y cultural del usuario y de sus familiares, contribuyendo a la reducción del riesgo de infecciones, de la recurrencia de hospitalizaciones y del uso inadecuado de los servicios hospitalarios. Además, favorece la optimización en la gestión eficiente de las camas hospitalarias y de los recursos de salud disponibles, sirviendo como una “puerta de salida” cualificada de la red de urgencias y emergencias, con potencial para disminuir la sobrecarga en estos servicios^3^.

El PMeC se centra en la deshospitalización de los usuarios que requieren seguimiento clínico y el soporte de un equipo multiprofesional, promoviendo no solo el cuidado, sino también acciones educativas dirigidas a usuarios, familiares, responsables y cuidadores para la continuidad de la asistencia en el domicilio. La actuación del PMeC se realiza a través de los Servicios de Atención Domiciliaria (SAD), los cuales son complementarios a la APS y a los servicios de urgencias, y también pueden ser sustitutivos de la hospitalización^2^.

Ante el envejecimiento poblacional y la demanda creciente de una atención de salud más integral y humanizada, los servicios de AD se han expandido en todo el mundo^4^. En el contexto brasileño, los SAD comenzaron a estructurarse con mayor intensidad a partir de 1990, siendo incorporados de forma estratégica y definitiva al SUS en 2011, con la creación del PMeC^5^.

Factores como la transición demográfica y epidemiológica acelerada, el aumento de los costos en salud, el proceso de desinstitucionalización y la búsqueda de modelos de cuidado más humanizados evidencian la importancia de la expansión del PMeC en el escenario nacional^3^ ^-^ ^5^. Para perfeccionar su gestión, resulta importante comprender las percepciones de los profesionales y gestores acerca del programa en cuanto a sus desafíos y potencialidades.

Los equipos multiprofesionales que actúan en el PMeC experimentan, en la práctica de la asistencia prestada a los usuarios, diferentes barreras y desafíos, siendo, por lo tanto, una fuente valiosa para la identificación de elementos que puedan sustentar mejoras en la conducción del programa. Sin embargo, a pesar de la relevancia de sus experiencias, no se identificaron, en la literatura científica, revisiones que reunieran las percepciones de los profesionales y gestores acerca del PMeC desde su creación. En este contexto, esta revisión tuvo como objetivo mapear en la literatura científica las potencialidades y fragilidades del Programa *Melhor em Casa* identificadas por profesionales de la salud y gestores.

## Método

### Tipo de estudio

Se trata de una revisión de la literatura basada en evidencias científicas, del tipo revisión de alcance, guiada por las recomendaciones del JBI *Reviewer’s Manual*, el cual orienta el mapeo de conceptos clave, la clarificación de áreas de investigación y la identificación de lagunas del conocimiento^6^. Para el desarrollo del estudio se recorrieron cinco etapas propuestas por el JBI: 1) Formulación de la pregunta de investigación; 2) Identificación de los estudios relevantes; 3) Selección de los estudios; 4) Extracción de los datos; y 5) Síntesis y presentación de los resultados. El protocolo de investigación fue registrado en la plataforma *Open Science Framework* bajo el DOI 10.17605/OSF.IO/6UCDB^7^. La extensión *Preferred Reporting Items for Systematic Reviews and Meta-Analyses for Scoping Reviews* (PRISMA-ScR) se utilizó para informar con transparencia la conducción de los resultados del análisis de alcance^8^. El estudio se realizó en la ciudad de Porto Alegre, en el estado de Rio Grande do Sul, Brasil, en el período de febrero de 2024 a marzo de 2025.

### Criterios de selección

Se consideraron como criterios de inclusión: estudios publicados íntegramente, disponibles electrónicamente, sin restricción de idioma, que respondieran a la pregunta de investigación propuesta. Se estableció como recorte temporal el período de 2011 a 2024, debido a la homologación de la Orden del PMeC por el Ministerio de Salud (MS) en 2011^9^, abarcando así trece años de implementación del programa en el territorio brasileño. Se excluyeron editoriales, cartas al editor, sitios web, noticias y resúmenes de eventos científicos.

### Recolección de datos

Para la estrategia de búsqueda de la revisión se adoptó la estructura mnemónica PCC, considerando: Población (P) - profesionales de la salud y gestores; Concepto (C) - potencialidades y fragilidades del PMeC; y Contexto (C) - Brasil. La pregunta formulada fue: “¿*Cuáles son las potencialidades y fragilidades observadas por los profesionales de la salud y gestores sobre el Programa Melhor em Casa en Brasil*?”

En la estrategia de búsqueda se incluyeron términos presentes en la estructura PCC; se utilizaron descriptores identificados en los tesauros del área de la salud, como *Medical Subject Headings* (MeSH) y Descriptores en Ciencias de la Salud (DeCS). Al tratarse de un programa exclusivo del contexto brasileño, la investigación contó con el apoyo de una bibliotecaria para la construcción de la estrategia de búsqueda, sugiriéndose utilizar el propio nombre del programa en portugués “*Programa Melhor em Casa*” y, en inglés, “*Better at home*”, con el fin de lograr una mayor cobertura bibliográfica sobre la temática y ampliar la estrategia de búsqueda (Figura 1).

La búsqueda de evidencias científicas se realizó en las siguientes fuentes de datos: Literatura Latinoamericana y del Caribe en Ciencias de la Salud (LILACS); *Public Medical Literature Analysis and Retrievel System Online* (PubMed/MEDLINE); Scopus; *Excerpta Medica data BASE* (EMBASE); *Web of Science* (WoS); y *Cumulative Index to Nursing and Allied Health Literature* (CINAHL). La literatura gris se investigó en la *Biblioteca Digital Brasileira de Teses e Dissertações* de la CAPES.

**Figura 1:** Estrategias de búsqueda y bases de datos utilizadas en la revisión de la literatura. Porto Alegre, RS, Brasil, 2025

Base de datos	Estrategia de búsqueda
LILACS*	(“melhor em casa” OR “programa melhor em casa”) AND programa AND (year_cluster:[2011 TO 2024]) AND ( db:(“LILACS”)) AND (year_cluster:[2011 TO 2024])
PubMed/MEDLINE	(“Better at home” OR “Better at home program”) AND program AND (brazil OR brazilian) Filters: from 2011 - 2024 (((“better”[All Fields] AND (“home environment”[MeSH Terms] OR (“home”[All Fields] AND “environment”[All Fields]) OR “home environment”[All Fields] OR “home”[All Fields])) OR (“better”[All Fields] AND (“home environment”[MeSH Terms] OR (“home”[All Fields] AND “environment”[All Fields]) OR “home environment”[All Fields] OR “programmable”[All Fields] OR “programmably”[All Fields] OR “programme”[All Fields] OR “programme s”[All Fields] OR “programmed”[All Fields] OR “programmer”[All Fields] OR “programmer s”[All Fields] OR “programmers”[All Fields] OR “programmes”[All Fields] OR “programming”[All Fields] OR “programmings”[All Fields] OR “programs”[All Fields]))) AND (“program”[All Fields] OR “program s”[All Fields] OR “programe”[All Fields] OR “programed”[All Fields] OR “programes”[All Fields] OR “programing”[All Fields] OR “programmability”[All Fields] OR “programmable”[All Fields] OR “programmably”[All Fields] OR “programme”[All Fields] OR “programmes”[All Fields] OR “programmed”[All Fields] OR “programmer”[All Fields] OR “programmers”[All Fields] OR “programmers”[All Fields] OR “programmes”[All Fields] OR “programming”[All Fields] OR “programmings”[All Fields] OR “programs”[All Fields]) AND (“brazil”[MeSH Terms] OR “brazil”[All Fields] OR “brazil”[All Fields] OR”home”[All Fields]) AND (“program”[All Fields] OR “programs”[All Fields] OR “programe”[All Fields] OR “programed”[All Fields] OR “programes”[All Fields] OR “programing”[All Fields] OR “programmability”[All Fields] OR “brazil”[All Fields] OR (“brazilian people”[Supplementary Concept] OR “brazilian people”[All Fields] OR “brazilians”[All Fields] OR “brazilian”[All Fields]))) AND (2011:2024[pdat])
Scopus	TITLE-ABS-KEY ( ( “Better at home” OR “Better at home program” ) AND program ) AND PUBYEAR > 2010 AND PUBYEAR < 2024
EMBASE	(‘better at home’ OR ‘better at home program’) AND ‘program’ AND [2011-2024]/py
WoS^†^	(“Better at home” OR “Better at home program”) AND program (All Fields) and 2018 or 2019 or 2023 or 2022 (Publication Years)
CINAHL	(‘better at home’ OR ‘better at home program’) AND ‘program’
CAPES *Catálogo de Teses e Dissertaçõe*s	“melhor em casa”

*LILACS = Literatura Latinoamericana y del Caribe en Ciencias de la Salud; ^†^WoS = *Web of Science*

### Selección de estudios

Todos los registros recuperados en las bases de datos fueron identificados, agrupados y cargados en la aplicación Rayyan^®^ (*Intelligent Systematic Review*), donde se eliminaron los duplicados y se realizó el cribado inicial de los títulos y resúmenes^10^. La literatura gris se analizó en hojas de cálculo de Excel^®^, también mediante lectura inicial del título y del resumen. Tras la eliminación de los duplicados en Rayyan^®^, se procedió a la etapa inicial de análisis de los datos; las revisoras realizaron la lectura independiente y ciega de los títulos y resúmenes de todos los documentos en la plataforma Rayyan^®^ y en la hoja de cálculo de Excel^®^. Las revisoras asignaron decisiones de aceptación o rechazo, de acuerdo con la pregunta de investigación de la revisión. El cegamiento de la plataforma Rayyan^®^ se abrió en una reunión entre las revisoras, con el fin de verificar las divergencias. En ese sentido, los estudios que permanecían con divergencias fueron evaluados por una tercera revisora, quien realizó la evaluación en la plataforma Rayyan^®^. En cuanto a la literatura gris, se realizó un encuentro presencial con las dos revisoras para evaluar los resultados y discutir las divergencias, de modo que no fue necesaria la intervención de una tercera revisora.

Los estudios seleccionados fueron leídos en su totalidad y analizados de manera descriptiva, de conformidad con la pregunta de investigación y con la relevancia del objetivo del estudio. En lo que respecta a la literatura gris, una disertación en particular no se encontró en su totalidad; se intentó contactar con el autor y la directora mediante correo electrónico sin éxito, y la otra disertación derivó de un artículo seleccionado en el estudio; por lo tanto, se optó por utilizar el artículo publicado.

### Tratamiento de los datos

Con base en los documentos seleccionados, los datos extraídos se organizaron en una hoja de cálculo de Excel^®^, que contenía información sobre: autor, título, tipo de estudio, año de publicación, idioma, muestra, revista de publicación, país de origen, año de publicación, objetivos del estudio, principales resultados y conclusiones. El análisis de los datos se realizó de forma descriptiva y, posteriormente, los hallazgos se categorizaron en potencialidades y fragilidades del PMeC mediante un análisis temático.

### Aspectos éticos

Al tratarse de una revisión de alcance, no fue necesaria la presentación del estudio al Comité de Ética en Investigación. Se afirma que no hubo conflicto de intereses por parte de las investigadoras involucradas y que se respetaron los derechos de autor de los documentos citados según la Ley nº 12.853 de 2013^11^.

## Resultados

En la búsqueda en las bases de datos, se identificaron 552 publicaciones. Tras la eliminación de 45 duplicados, quedaron 507 estudios. A continuación, se procedió a la lectura de los títulos y resúmenes, aplicándose los criterios de inclusión y exclusión por dos revisores independientes, lo que resultó en la selección de 27 estudios. Posteriormente, se realizó la lectura completa de los documentos seleccionados, considerando los criterios de inclusión y exclusión establecidos, culminando en una muestra final constituida por ocho publicaciones (Figura 2).

**Figura 2:** Diagrama de flujo PRISMA-ScR^12^ utilizado para la identificación y selección de estudios. Porto Alegre, RS, Brasil, 2025

El idioma principal de las publicaciones fue el portugués (n=5; 62,5%), seguido del inglés (n=3; 37,5%). En cuanto al diseño metodológico utilizado por los autores, predominaron los estudios con enfoque cualitativo (n=6; = 75%), seguidos por el enfoque cuantitativo (n=1;12,5%) y los métodos mixtos (n=1; 12,5%).

En los estudios, se identificaron 90 participantes. En relación con el año de publicación, se identificó que los artículos fueron publicados entre 2016 y 2022, siendo el mayor número en el año 2022 (n=3; 37,5%), seguido por los años 2018 (n=2; 25%), 2020 (n=1;12,5%), 2019 (n=1;12,5%) y 2016 (n=1; 12,5%). La Figura 3 presenta la caracterización de los estudios incluidos en la revisión, con información sobre autoría, título, tipo de estudio, año de publicación y número de participantes.

**Figura 3:** Caracterización de los estudios identificados en la revisión. Porto Alegre, RS, Brasil, 2025

Estudio	Autor	Título	Tipo de estudio	Año de publicación	Muestra (n=)
E1^13^	Verdi DS, Pires RRC	*Between standardization and flexibility: the implementation of federal program Better at Home considering local diversity*	Estudio descriptivo con enfoque cualitativo	2022	6
E2^14^	Almeida MB, Albuquerque IMAN, Silva MAM, Mayorga FDO, Balica HLL, Nascimento ABO	*Limits and Potentials of Pediatric Home Care in a Ceará State municipality*	Estudio descriptivo con enfoque cualitativo	2022	-
E3^15^	Cavalcante MEPL, Santos MM, Toso BRGO, Vaz EMC, Lima PMVM, Collet N	*It is better at home: characterization of home care services*	Estudio exploratorio cuantitativo	2022	23
E4^16^	Castro EAB, Leone DRR, Santos CM, Neta FCCG, Gonçalves JRL, Contim D, Silva KL	*Home care organization with the Better at Home Program*	Estudio descriptivo, exploratorio con enfoque cualitativo	2018	18
E5^17^	Lima ACB	*Análise da implantação do serviço de atendimento domiciliar na óptica dos profissionais da atenção primária à saúde*	Estudio descriptivo, exploratorio con enfoque cualitativo	2020	14
E6^18^	Maldonado TCP	*“Melhor em Casa!?” A resiliência do profissional frente a prática do atendimento domiciliar*	Estudio de casos múltiples con enfoque cualitativo	2019	9
E7^19^	Oliveira AV Neto	*Análise do Programa Melhor em Casa: um olhar sobre a atenção domiciliar no âmbito do Sistema Único de Saúde*	Estudio descriptivo, exploratorio cuantitativo y con enfoque cualitativo	2016	6
E8^20^	Canuto KF	*Melhor em Casa: um estudo de caso sobre a interdisciplinaridade no programa no município de Palmeira dos Índios, Alagoas*	Estudio evaluativo con enfoque cualitativo	2018	14

La Figura 4 sintetiza los objetivos de los estudios y los principales resultados de cada estudio, lo que permite visualizar las percepciones de los profesionales acerca del PMeC.

**Figura 4:** Síntesis descriptiva de los estudios incluidos en la revisión de alcance. Porto Alegre, RS, Brasil, 2025

Número	Objetivos	Principales percepciones de los profesionales
E1^13^	Conocer las diferentes experiencias de gestores locales con las directrices y normativas federales relativas a la implementación del Programa *Melhor em Casa*.	Como potencialidades, se observa la orden orientadora del programa como adecuada a la realidad. Se percibe la importancia de instrumentos de coordinación federal con adaptaciones locales, y la necesidad de flexibilización en las recomendaciones de la orden del PMeC*. La adhesión al programa aportó mayores recursos financieros a la AD^†^, adecuando el equipo de salud y el flujo asistencial. El PMeC* permitió que las defunciones ocurran en el domicilio y proporciona la deshospitalización. Sin embargo, aún se observa resistencia por parte del equipo médico hospitalario para derivar pacientes al programa, además de dificultades en la conformación de los equipos de AD^†^.
E2^14^	Conocer los límites y potencialidades de la atención domiciliaria pediátrica del Programa *Melhor em Casa* en un municipio del estado de Ceará.	La falta de conocimiento de los profesionales de diferentes niveles asistenciales sobre la AD^†^ perjudica la continuidad del cuidado; asimismo, se menciona la ausencia de algunas categorías profesionales, la falta de estructura física exclusiva para el PMeC* y la deficiencia de insumos esenciales para los cuidados. Se evidenció la carencia de transporte, la falta de capacitaciones, la ausencia de vínculos entre el programa y la gestión de la APS^‡^. Otra característica negativa mencionada fue el cambio de profesionales en cada ciclo de gestión, cortando el vínculo entre los profesionales, los pacientes y la red. No obstante, como un criterio de mejora se señala la disminución de internaciones hospitalarias pediátricas.
E3^15^	Caracterizar los Servicios de Atención Domiciliaria en funcionamiento en Paraíba.	El uso de la teleatención se presenta como una gran estrategia de cuidado para los usuarios. Como puntos susceptibles de mejora, se encuentra el transporte del SAD^§^, el cual se comparte con otros servicios de la red. Además, algunos equipos no cumplen la carga horaria mínima exigida, actúan en algunos turnos, lo que repercute en la continuidad de la asistencia. Las sedes del SAD^§^ suelen estar anexas a otros servicios de salud; aun con la interconexión, se produce fragmentación en la comunicación entre los servicios. En lo que respecta a la historia clínica electrónica del paciente y al PTS^||^, se realizan solo en el 50% de los servicios, evidenciándose además la fragmentación en algunos puntos esenciales para el conocimiento de la información de los pacientes y la planificación del cuidado individualizado.
E4^16^	Comprender la organización de la Atención Domiciliaria en el contexto de la atención a la salud pública ofrecida por municipios que se adhirieron al Programa *Melhor em Casa* en el Estado de Minas Gerais.	Perciben la integración con la red como una fragilidad del PMeC*. Realizar reuniones entre equipos de diferentes servicios y organizar procesos se consideran estrategias eficaces para mejorar la atención al usuario en la AD^†^.
E5^17^	Analizar cómo ocurrió el proceso de implantación del Servicio de Atención Domiciliaria desde la perspectiva de los profesionales de la atención primaria a la salud del municipio de Araranguá/Santa Catarina.	Se evidenció la importancia del PMeC* en la deshospitalización de pacientes, en la mejora de la calidad de vida de los usuarios y sus familiares, siendo una referencia para la APS^‡^ y el ámbito hospitalario. Como puntos de mejora, se presentan la falta de compartición de cuidados, la dificultad de interacción entre profesionales y el desconocimiento de derivaciones por parte de la APS^‡^. Además, existe sobrecarga del equipo SAD^§^, falta de estructura física y de vehículos, y dificultad de responsabilización del usuario por el cuidado. Se propone como sugerencia la educación permanente en salud con los profesionales de la RAS^¶^.
E6^18^	Comprender la experiencia de profesionales del Programa *Melhor em Casa* a la luz de la resiliencia.	El PMeC* se percibe como un servicio acogedor, resolutivo, que realiza un trabajo multiprofesional humanizado, con una mirada más allá de las demandas clínicas. La atención abarca a las familias con apoyo y acogida, convirtiéndose en un servicio diferenciado en la RAS^¶^. El servicio ingresa en los hogares y, por tener un contacto tan íntimo con situaciones diversas, los profesionales están expuestos a sobrecarga, impotencia y fragilidad. Debido al gran vínculo con las familias, algunas manifiestan temor de recibir el alta del PMeC*.
E7^19^	Caracterizar la implantación del Programa *Melhor em Casa* y analizarlo desde la perspectiva de los gestores municipales	El PMeC* se percibe como una alternativa a la internación hospitalaria, con gran potencial de deshospitalización y articulación con la RAS^¶^. Optimiza camas hospitalarias, se inserta en la red para discutir casos y compartir cuidados. Debe prestarse mayor atención a la divulgación del programa por parte del Ministerio de Salud a la población, siendo necesaria una mayor explicación de la orden junto con la RAS^¶^, a fin de mejorar el entendimiento. Se requieren incentivos para incrementar la calidad técnica del equipo del PMeC* y el suministro adecuado de insumos y capacitaciones a la APS^‡^ para la derivación adecuada de pacientes.
E8^20^	Evaluar la comprensión de los profesionales sobre la interdisciplinariedad en las acciones del Programa *Melhor em Casa* en el Municipio de Palmeira dos Índios/Alagoas.	La interdisciplinariedad aún tiene un significado poco conocido o definido de forma equivocada entre los profesionales. En el SAD^§^, ocurre mediante acciones en el día a día del servicio, en relaciones horizontales con intercambios o atención compartida. El trabajo en equipo está íntimamente ligado; la falta de vehículos exclusivos para el SAD^§^, o el uso de un vehículo exclusivo para curaciones, demuestra la falta de compartición del cuidado. Las admisiones son realizadas solo por un profesional de trabajo social, sin que se realicen intercambios, y no se realiza el PTS^||^. Se evidencia la necesidad de mejoras en la relación interpersonal, encuentros de equipo, atención multiprofesional, así como la divulgación de los criterios del programa para la RAS^¶^.

*PMeC = Programa *Melhor em Casa*; ^†^AD = Atención Domiciliaria; ^‡^APS = Atención Primaria de Salud; ^§^SAD = Servicio de Atención Domiciliaria; ^||^PTS = Plan Terapéutico Singular; ^¶^RAS = Red de Atención a la Salud

Para favorecer la comprensión y la organización de los resultados, así como responder a la pregunta de investigación de la revisión, los hallazgos se subdividieron en dos categorías temáticas: Potencialidades y Fragilidades del PMeC, según la Figura 5.

**Figura 5:** Categorías temáticas de la revisión de alcance. Porto Alegre, RS, Brasil, 2025

Categorías temáticas
Potencialidades del Programa Melhor em Casa
Construcción del trabajo en red^13^ ^-^ ^14^ ^),(^ ^16^ ^),(^ ^18^ ^-^ ^20^; Integración del servicio con la sociedad^13^; Protocolos y procesos organizacionales^16^; Orden orientadora adecuada a la realidad^13^; Deshospitalización y reducción de internaciones innecesarias^13^ ^-^ ^14^ ^),(^ ^17^; Educación en salud con las familias^14^ ^),(^ ^16^ ^-^ ^17^; Teleatención^15^; Plan Terapéutico Singular (PTS)^16^; Flexibilidad y autonomía por parte de la gestión^20^; Humanización^15^; y Mejora en la calidad de vida de los usuarios^17^.
**Fragilidades del Programa Melhor em Casa**
Educación permanente^14^ ^-^ ^20^; Interlocución con la RAS*^13^ ^-^ ^19^; Estructuración de los servicios^13^ ^-^ ^17^ ^),(^ ^20^; Responsabilización del usuario por el cuidado^19^ ^-^ ^20^; Desgaste emocional de los profesionales^18^ ^-^ ^19^; Falta de recursos tecnológicos^15^; Uso de la historia clínica electrónica^15^; y Fragmentación del equipo^20^.

*RAS = Red de Atención a la Salud

## Discusión

Los hallazgos de esta revisión de alcance evidenciaron dos categorías temáticas que caracterizan la actuación del programa en el escenario brasileño: potencialidades y fragilidades del PMeC. El análisis crítico permite comprender los avances alcanzados y los desafíos persistentes en la consolidación de la AD como estrategia integrada a la RAS.

### Potencialidades para el Programa *Melhor em Casa*

La construcción del trabajo en red, como eje estructurante del PMeC, se destacó como la principal potencialidad evidenciada en los estudios analizados. La actuación articulada entre los distintos puntos de la RAS se muestra fundamental, considerando que el programa atiende, mayoritariamente, a usuarios procedentes de internaciones hospitalarias recientes. Tal configuración demanda la articulación con la red intrahospitalaria para garantizar la transición segura del cuidado, el establecimiento de vínculo intrafamiliar del equipo del PMeC en el domicilio y, posteriormente, la integración del usuario en la APS, componiendo un continuo asistencial en la RAS. En este contexto, la consolidación del trabajo en red se hace necesaria y está intrínsecamente relacionada con la estructura del servicio, contribuyendo a la obtención de mejores desenlaces clínicos. Una revisión narrativa destacó que la calidad de la atención domiciliaria está influida tanto por la capacidad de los profesionales para acoger las necesidades como por el establecimiento de una comunicación directa con la familia^21^. Esto refuerza la articulación con los diferentes niveles de la RAS, lo que favorece la formulación de estrategias para acciones integradas que atiendan, de manera multidisciplinaria, las necesidades y expectativas del usuario y su familia frente a las incertidumbres asociadas al cuidado domiciliario.

Otro punto evidenciado en los estudios se refiere a la deshospitalización y a la reducción de internaciones consideradas evitables, corroborando los objetivos centrales del programa, que apuntan a la transición segura de usuarios estables al domicilio. El retorno del usuario al entorno domiciliario se configura como una acción de continuidad del cuidado iniciado en el contexto hospitalario y, cuando se realiza de forma planificada y con acceso a los recursos necesarios, permite mantener la articulación con los diferentes niveles de la red, de manera integrada, resolutiva y centrada en la persona. La programación de un alta segura implica la evaluación de las necesidades clínicas y sociales del usuario, así como de la estructura familiar y de la disponibilidad de servicios de la RAS, elementos fundamentales para la prevención de reinternaciones^22^. Aún en este contexto, la reducción de las internaciones innecesarias se ve favorecida por el reconocimiento precoz de cambios en el cuadro clínico del usuario y por la conducción adecuada de la atención a las intercurrencias, siendo esto resultado del proceso educativo promovido por los equipos junto a las familias, lo que contribuye a disminuir el retorno del usuario al entorno hospitalario.

La educación en salud dirigida a las familias fue representada en los estudios como una de las estrategias diferenciales de la actuación del PMeC. El programa trasciende el enfoque en la deshospitalización al invertir en acciones educativas con cuidadores y familiares en el espacio domiciliario, entorno en el que ocurren intensos intercambios de saberes. En este contexto íntimo y singular, marcado por la reorganización de la rutina familiar ante la presencia de un familiar enfermo, es esencial que el equipo demuestre empatía y compromiso para, de manera progresiva, promover la construcción de conocimientos entre los familiares para el ejercicio de un cuidado seguro e integral. La educación en salud debe, por lo tanto, concebirse como una herramienta para la promoción de la autonomía y el protagonismo de todos los involucrados, incentivando la corresponsabilización en el proceso de cuidado^23^.

La adopción de la teleatención y del PTS también se destacó como una estrategia que personaliza el cuidado, situando al usuario como agente central de su proceso de rehabilitación y promoviendo mayor agilidad y efectividad en las acciones asistenciales. La teleatención se constituye en un sistema de apoyo que permite al usuario permanecer en casa, así como los recursos visuales de la plataforma posibilitan el desarrollo de relaciones interpersonales con los profesionales de la salud a lo largo del tiempo. La modalidad es una alternativa viable para monitorear síntomas, orientar acciones e incentivar la adhesión al cuidado domiciliario, explorando posibilidades tanto asistenciales como educativas^24^. En lo que se refiere a la planificación del cuidado, el PTS se presenta como un instrumento que sirve de guía para las acciones del equipo multiprofesional, siendo compartido entre los miembros del equipo, con miras a la implementación de intervenciones alineadas a las necesidades singulares de cada usuario^25^ ^-^ ^26^.

### Fragilidades para el Programa *Melhor em Casa*

Las fragilidades más recurrentes identificadas en los estudios fueron la educación permanente y la interlocución con la RAS, evidenciando dos aspectos centrales que demandan atención cualificada en el contexto nacional. Otro aspecto señalado con frecuencia en los estudios se refiere a la estructura de los servicios, seguido de la responsabilización del usuario por el cuidado y del desgaste emocional de los profesionales involucrados en la AD.

La educación permanente, reconocida como una estrategia de enseñanza-aprendizaje en servicio, con principios problematizadores, interdisciplinares e interprofesionales en un contexto real, se centra en las necesidades cotidianas de la práctica profesional para la cualificación de la asistencia prestada al usuario, especialmente en el PMeC, dada la complejidad del cuidado domiciliario. Este proceso educativo ha proporcionado transformaciones graduales en las prácticas de salud, con el propósito de alcanzar la mejora del cuidado, la reorganización del proceso de trabajo y la calidad de la gestión pública, mediante acciones pedagógicas innovadoras y alineadas al modelo asistencial vigente^27^. Tales acciones deben contemplar metodologías innovadoras, incluida la enseñanza virtual y recursos tecnológicos, que favorezcan el desarrollo de competencias de los trabajadores de la salud^4^.

La fragmentación en la interlocución con la RAS o las dificultades de acceso en situaciones de intercurrencia con los usuarios en el PMeC configuran obstáculos relevantes que conducen al desgaste emocional en los equipos multiprofesionales, así como afectan la capacidad resolutiva de las necesidades del usuario. Estas barreras comprometen la integralidad de la atención, pudiendo ocasionar discontinuidad del cuidado, recurrencia de agravios de salud y reinternaciones evitables, frecuentemente relacionadas con la fragilidad de la articulación entre los servicios que componen la red. La integración efectiva entre los servicios de la RAS, con base en una visión compartida, adopción de directrices clínicas y organización horizontal, es imperativa para la cualificación del cuidado y la reducción de redundancias^28^.

La estructuración de los servicios, a su vez, abarca desde limitaciones en instalaciones físicas inadecuadas hasta la insuficiencia de vehículos, frecuentemente compartidos con otros servicios o unidades del municipio, comprometiendo la realización de las visitas domiciliarias. La oferta adecuada de recursos materiales, aunque esencial, no es suficiente para asegurar la efectividad de la asistencia en el contexto domiciliario. Para que la AD se realice de manera eficiente y segura, se requiere una planificación estratégica que incorpore la educación permanente y garantice la asignación adecuada de recursos materiales y una gestión de personas cualificada^28^.

Otro aspecto emergente en los estudios se refiere a la necesidad de incorporar nuevas tecnologías, como el PEP. La ausencia de este recurso limita el intercambio de información entre los servicios de la RAS, dificultando la continuidad y la integralidad del cuidado. La implementación del PEP, además de proporcionar mayor practicidad y agilidad en la gestión de la información, es esencial para la expansión de la AD^29^. El PEP contribuye a la seguridad y la eficacia de la atención, al permitir el acceso al historial del usuario y sustentar la toma de decisiones clínicas^26^.

La responsabilización del usuario y de la familia por el cuidado constituye un desafío en algunos estudios. Este aspecto requiere abordaje desde la admisión del usuario en el programa, mediante pactaciones claras con la familia respecto de los procesos de cuidado y del alta, asegurando que usuarios y cuidadores estén instrumentalizados para el cuidado, de modo que despierte interés, corresponsabilidad e intercambio de saberes y conocimientos^30^. Es necesario promover la autonomía de la familia en el proceso de corresponsabilización e incentivar el protagonismo de los involucrados, y consolidar la AD como una referencia para el cuidado siempre que sea necesario^31^. La AD se configura como una nueva alternativa innovadora de producción del cuidado fuera de las instituciones tradicionales, como los hospitales, permitiendo que la familia y el cuidador asuman un papel central en la gestión del cuidado en el domicilio, contribuyendo a la construcción de estrategias más adecuadas a las realidades del territorio^32^.

Otro aspecto de vulnerabilidad identificado en dos estudios fue el desgaste emocional de los profesionales, especialmente en el cuidado de usuarios con enfermedades crónicas. La actuación en el domicilio expone al profesional a realidades adversas, incluidas condiciones precarias de vivienda y escasez de recursos, condiciones que potencian el sufrimiento psíquico y la sobrecarga emocional. Tales condiciones exigen la atención de los gestores respecto de la salud mental de los equipos, mediante acciones de educación en salud, espacios de escucha y apoyo psicosocial^33^. La exposición continua a riesgos psicosociales puede resultar en impactos físicos, mentales y sociales significativos, con frecuencia desatendidos por prejuicios que asocian el sufrimiento psíquico a la fragilidad individual, en detrimento de la comprensión de las condiciones laborales involucradas en el quehacer cotidiano en la AD^34^.

Entre las limitaciones del estudio, se destaca la escasa disponibilidad de producciones científicas sobre la temática, lo que evidencia la necesidad de investigaciones adicionales que permitan conocer de forma más amplia el potencial del programa e identificar mejoras que puedan aplicarse al PMeC. Considerando las diversidades culturales y geográficas del país, muchas acciones pueden adaptarse, recrearse y expandirse. La educación permanente también se presenta como una limitación relevante, indicando la pertinencia de desarrollar estudios de intervención orientados a la cualificación de los profesionales en el PMeC.

Las contribuciones de este estudio para la enfermería son significativas, al ofrecer un análisis crítico y amplio del PMeC, destacando sus potencialidades y fragilidades en el contexto de la AD integrada a la RAS. La actuación de la enfermería en el contexto del equipo multiprofesional en el PMeC se revela esencial en la articulación del cuidado en red, en la educación en salud a las familias y en la promoción de la continuidad asistencial segura y centrada en el usuario. Por otro lado, el estudio sustenta el perfeccionamiento de las prácticas profesionales y contribuye al fortalecimiento de la AD como estrategia innovadora y resolutiva en lo que respecta a la educación permanente, el uso de tecnologías como la teleatención y la historia clínica electrónica, y la valorización del cuidado de la salud mental frente al desgaste emocional de los equipos. De este modo, se refuerza el papel estratégico de la enfermería en la coordinación del cuidado, en la cualificación de la atención prestada en el domicilio y en la construcción de modelos asistenciales más sostenibles, eficientes y humanizados.

## Conclusión

El mapeo de las producciones científicas sobre las potencialidades y fragilidades del PMeC, identificadas por profesionales de la salud y gestores, resultó limitado en cantidad en comparación con otras áreas de conocimiento en salud. Como potencialidades del PMeC, se identificaron la construcción del trabajo en red, la deshospitalización, la reducción de reinternaciones evitables, la educación en salud junto a las familias, la teleatención, la utilización del PTS, la flexibilidad y autonomía en la gestión, así como la humanización y la mejora de la calidad de vida de los usuarios. En lo que se refiere a las fragilidades observadas, se destacan aspectos que demandan medidas cualificadas para el perfeccionamiento del programa y de la atención a la salud en el ámbito domiciliario: la educación permanente, la interlocución con la RAS, la estructura de los servicios, la corresponsabilización del usuario y de los familiares por el cuidado, el desgaste emocional de los profesionales, la insuficiencia de recursos tecnológicos, la ausencia del PEP y la fragmentación del equipo.

De este modo, el estudio evidencia la relevancia del PMeC en el sistema de salud brasileño, al demostrar su potencial para cualificar la atención domiciliaria. Para ello, se requieren intervenciones estratégicas e inversiones estructurales que fortalezcan el programa y aseguren su efectividad y sostenibilidad a largo plazo. Se recomienda, además, la realización de nuevos estudios que profundicen los temas abordados, contribuyendo al avance del conocimiento y al perfeccionamiento de las prácticas en el ámbito de la AD ofrecida por el PMeC.

## Data Availability

Todos los datos generados o analizados durante este estudio están incluidos en este artículo publicado.
